# Putative new combination vaccine candidates identified by reverse vaccinology and genomic approaches to control enteric pathogens

**DOI:** 10.1186/s12865-024-00626-y

**Published:** 2024-07-22

**Authors:** Saeed Mikaeel, Abbas Doosti, Ali Sharifzadeh

**Affiliations:** 1 Department of Biology, Faculty of Basic Sciences, Shahrekord Branch, Islamic Azad University, Shahrekord, Iran; 2 Biotechnology Research Center, Shahrekord Branch, Islamic Azad University, Shahrekord, Iran; 3 Department of Microbiology, Faculty of Veterinary Medicine, Shahrekord Branch, Islamic Azad University, Shahrekord, Iran

**Keywords:** Multi-epitope vaccine, Structural vaccinology, Linker, Intestinal disease Bioinformatics

## Abstract

**Objectives:**

The pathogenic microorganisms that cause intestinal diseases can significantly jeopardize people's health. Currently, there are no authorized treatments or vaccinations available to combat the germs responsible for intestinal disease.

**Methods:**

Using immunoinformatics, we developed a potent multi-epitope Combination (combo) vaccine versus Salmonella and enterohemorrhagic E. coli. The B and T cell epitopes were identified by performing a conservancy assessment, population coverage analysis, physicochemical attributes assessment, and secondary and tertiary structure assessment of the chosen antigenic polypeptide. The selection process for vaccine development included using several bioinformatics tools and approaches to finally choose two linear B-cell epitopes, five CTL epitopes, and two HTL epitopes.

**Results:**

The vaccine had strong immunogenicity, cytokine production, immunological properties, non-toxicity, non-allergenicity, stability, and potential efficacy against infections. Disulfide bonding, codon modification, and computational cloning were also used to enhance the stability and efficacy of expression in the host E. coli. The vaccine's structure has a strong affinity for the TLR4 ligand and is very durable, as shown by molecular docking and molecular modeling. The results of the immunological simulation demonstrated that both B and T cells had a heightened response to the vaccination component.

**Conclusions:**

The comprehensive in silico analysis reveals that the proposed vaccine will likely elicit a robust immune response against pathogenic bacteria that cause intestinal diseases. Therefore, it is a promising option for further experimental testing.

**Supplementary Information:**

The online version contains supplementary material available at 10.1186/s12865-024-00626-y.

## Introduction

Enteric diseases continue to be a significant cause of sickness and mortality in young children in poor countries [1]. Pathogens that cause gastrointestinal diseases include parasitic, viral, and bacterial species. Bacterial pathogens are prevalent among the causative agents of gastrointestinal diseases, making them noteworthy. Salmonella and enterohemorrhagic E. coli are the primary culprits behind enteric infections [1, 2]. The existing state of things described here has implications for developing enteric vaccines. Economic factors often serve as the primary motivators for vaccine research [1, 2].

The development of vaccinations to combat uncommon diseases in industrialized nations often takes precedence, leading to a neglect of diseases prevalent in developing countries. For instance, our progress in developing vaccines for eukaryotic infections has been limited. The challenge of developing effective vaccines is further complicated by the fact that these diseases primarily affect the gut. Traditional parenteral injection approaches may not be the most effective strategy to generate immunity against a gut-colonizing pathogen, as they primarily stimulate systemic immune reactions. This highlights the need for innovative approaches to induce specific immune responses in the stomach for more effective protection [1, 2].

*Salmonella enterica serovar Typhimurium* is the primary culprit behind gastrointestinal diseases in people and animals. It thrives when contaminated food or water penetrates the intestinal epithelium and causes illness [3].

Research has shown that the infection spreads by post-dissemination, invasion of phagocytes, and invasion of the intestinal epithelium in many individuals. S. typhimurium, often known as non-typhoid Salmonella, is a prevalent bacterium transmitted by food that may lead to several types of diarrheal illnesses [4]. Common symptoms of this condition include pyrexia, gastrointestinal disturbances, abnormally liquid faeces, and abdominal discomfort. In countries with insufficient sanitation, S. typhimurium may lead to life-threatening invasive diseases such as meningitis, sepsis, and bacteremia. Individuals who are children, old, or have compromised immune systems are more vulnerable to the illnesses caused by this bacterium. S. typhimurium is a pathogenic bacterium that threatens human health [5].

*E. coliO157:H7 has* emerged as a significant, global food-borne pathogen that may cause serious illnesses such as hemolytic uremic syndrome (HUS) and thrombotic thrombocytopenic purpura (TTP) [6]. According to an epidemiological analysis, Shiga toxin enterohemorrhagic diarrhoea epidemics have been linked to domesticated animals, notably feedlot cattle, which have identified cattle as the primary reservoir for *E. coliO157: H7*[7]. Farmyard ruminants serve as a natural reservoir for *E. coli*0157: H7. An estimated 63,000 cases of hemorrhagic colitis are caused by *E. coliO157:H7* per year in the US [8]. According to a database review and research from 10 of the subregions of the World Health Organization, there are 2.8 million cases of *E. coli* worldwide each year.

Specifically, *E. coliO157:H7*-induced HUS may cause systemic morbidities such as acute renal failure in children. Shiga toxin, produced by *E. coliO157:H7*, damages the gut by sloughing off intestinal mucosa cells and causes hemorrhagic diarrhoea, which is the pathophysiology of this bacteria [8]. The Shiga toxin presents as a hemolytic uremic syndrome, stomach discomfort, and, in rare cases, thrombotic thrombocytopenic purpura due to its systemic effects on vascular endothelial cells, which cause vasculitis. As the renal glomeruli are particularly vulnerable to the microthrombi formation, *E. coli*0157:H7 Shiga toxin starts the inflammatory cascade that results in leukocyte aggregation, death of the afflicted cells, platelet aggregation, microthrombi development, hemolysis, and renal failure [9, 10]. Most of these gastrointestinal pathogens are conveyed through faeces to the mouth. Infections are often brought on by consuming contaminated food or drink, which may result in illnesses like nausea, cramping in the abdomen, and fever. E. coli O157:H7 is a prominent food-borne pathogen with a worldwide impact, capable of causing severe disorders such as hemolytic uremic syndrome (HUS) and thrombotic thrombocytopenic purpura (TTP) [6]. Based on an epidemiological investigation, Shiga toxin enterohemorrhagic diarrhoea outbreaks have been associated with domesticated animals, particularly feedlot cattle. These cattle have been identified as the primary E. coli O157: H7 bacteria source. Farmyard ruminants provide as a natural source for E. coli0157: H7. Approximately 63,000 cases of hemorrhagic colitis are attributed to E. coli O157:H7 annually in the United States [8]. Based on a comprehensive analysis of a database and research conducted in 10 subregions of the World Health Organization, it has been determined that about 2.8 million cases of E. coli are reported globally annually. More precisely, the development of Hemolytic Uremic Syndrome (HUS) caused by the E. coliO157:H7 bacteria may lead to many health complications, including acute renal failure, particularly in youngsters. Shiga toxin, produced by the bacterium E. coliO157:H7, causes damage to the gastrointestinal tract by shedding the intestinal mucosa cells, resulting in hemorrhagic diarrhoea. This is the underlying mechanism of action of this particular strain of bacteria [8]. The Shiga toxin manifests as hemolytic uremic syndrome, abdominal pain, and, in rare instances, thrombotic thrombocytopenic purpura due to its systemic impact on vascular endothelial cells, leading to vasculitis. Due to their susceptibility to the creation of microthrombi, the renal glomeruli are significantly affected by the E. coli0157:H7 Shiga toxin. This toxin initiates an inflammatory response that leads to the aggregation of white blood cells, death of the affected cells, aggregation of platelets, development of microthrombi, destruction of red blood cells, and, ultimately, renal failure [9, 10]. Most gastrointestinal infections are transmitted by faecal matter to the oral cavity. Infections are often caused by ingesting contaminated food or drink, leading to symptoms such as nausea, abdominal cramps, fever, and, most commonly, diarrhoea and dehydration [11]. Enteric infections often resolve spontaneously in healthy adults. However, they may progress to life-threatening conditions and can lead to death in young infants and individuals with weakened immune systems if medical care is not provided [12]. Enteric infections heal on their own in healthy people, but in small children and those with weakened immune systems, the infection may progress to a severe illness that can be fatal if left untreated [12].

Implementing comprehensive sanitation systems and ensuring access to clean water may effectively prevent enteric infections. Access to water, sanitation, and hygiene (WASH) is challenging for many countries that lack sufficient resources [13]. Using soap, especially antibacterial soap, reduces enteric infections in the short term and increases the risk of enteric microorganisms acquiring resistance to antimicrobial agents [14]. On the other hand, immunization is considered to be a more efficient and feasible method for avoiding enteric illnesses. Regrettably, various microorganisms are associated with gastrointestinal disorders, but no authorised immunisations exist for these diseases [15]. The challenges in designing effective vaccines against enteric infections include the heterogeneity of individual pathogen strains (genotypes, serotypes, or pathotypes), the unavailability of appropriate animal models for evaluating vaccine efficacy in human studies, limited understanding of pathogen pathogenesis or disease mechanisms (such as nontyphoidal Salmonella), the absence of defined host immune markers for protection, the short duration of protective immunity, and the lack of suitable animal models. The primary obstacle is the incapacity to develop universally efficacious vaccines for several gastrointestinal illnesses [15, 16].

In addition, patients with gastrointestinal diseases often harbour many microorganisms in their bodies [17]. This poses a more significant challenge in diagnosing and treating illnesses, which requires the development of combination vaccines that may protect against two or more intestinal infections. As more vaccines continue to be released, the current WHO EPI (expanded program for immunization) is getting increasingly crowded, which makes combination (combo) vaccines more attractive [18].

This study focuses on developing innovative multi-epitope vaccine candidates using Reverse vaccinology. The aim is to design vaccines specifically for enterohemorrhagic E. coli (EHEC) and Salmonella, the leading causes of enteric infections. Additionally, we introduce a new vaccine technology or vaccinology platform that enables the development of cross-protective multivalent vaccines.

## Materials and methods

### Protein sequence retrieval

The amino acid sequence of the primary proteins of Salmonella and enterohemorrhagic E. coli (EHEC) was obtained using the vaxign database, which may be accessed at https://violinet.org/vaxign2. Moreover, the proteins' accession IDs were obtained via the search engine inside the search box of the UniProt datasets.

### Prediction of linear B lymphocyte (LBL) epitope

We used the ABCpred program (http://crdd.osdd.net/raghava/abcpre/ABC submission.html) to forecast linear B cell epitopes. When given a FASTA sequence as input, the program generates 10mer epitopes for B-cell ligands, with a default specificity of 75%. The software uses a window length of 10 to analyze the sequence. In the future, producing a multi-epitope vaccine will prioritize the epitope with the highest score [19–21].

### Prediction of cytotoxic T lymphocyte (CTL) epitope

The NetMHCpan4.1 website was utilized to predict the target protein's CTL epitopes. The server may be accessed at http://www.cbs.dtu.dk/services/NetMHCpan-4.1/. The epitope identification threshold was established at 0.5% for a robust binding agent and 2% for a weak binding agent. The site uses predictive algorithms to identify epitopes specific to HLA class I supertypes. The epitope of each supertype was determined. The decision was made to arrange the data based on the prediction score. The epitope lengths were pre-determined to be 9mers, conserved epitopes that bind to many HLA alleles [20–22].

### Epitope prediction for helper T lymphocytes (HTL)

The selected protein was inputted into the MHC II binding algorithm of the IEDB (Immune Epitope and Database) to predict (15mer) HTL (helper T lymphocyte) epitopes. The whole HLA (Human Leukocyte and Antigen) reference collection was employed to forecast the HTL epitopes. Every HLA class II allele, such as HLA-DR, HLA-DQ, and HLA-DP, is included. A recommended factor in making the forecast was a comprehensive collection of 27 human alleles that included 99% of the population. The window size remained constant at 15, and the peptides were organized in ascending order based on the adjusted rank. The selection of HTL epitopes was based on their IC50 values, with epitopes having low IC50 values demonstrating high affinity. The IC50 value represents the minimum dose of a medication needed to achieve 50% inhibition in vitro. This selection process was supported by references [23–25].

### Evaluation of toxicity, allergenicity, and antigenicity

Antigenicity was predicted using the Vaxijen v2.0 service, available at http://www.ddg-pharmfac.net/vaxijen/VaxiJen/VaxiJen.html. This software requires an epitope sequence to accurately categorize an antigen by its specific physicochemical properties. The antigenicity of the epitope sequence was forecasted utilizing the default settings (threshold = 0.4, ACC output) compared to the microbial genus [21, 26].

The bioinformatics tool AllerTOP v. 2.0 was used to assess the allergenic potential of epitopes derived from bacterial protein sequences and their ability to induce allergy reactions in individuals. The allergenicity of the sequence [22, 26] was determined by using all of the epitopes in the plain text as input.

The toxicity of specific epitopes was forecasted utilizing ToxinPred, accessible at https://webs.iiitd.edu.in/raghava/toxinpred/multi/submit.php. The peptides were supplied in the FASTA format, and the default settings were utilized for all other parameters [23, 27]. The immunogenicity of the chosen peptides was determined using Class I Immunogenicity from the IEDB Analysis Source, which may be found at http://tools.iedb.org/immunogenicity. The epitopes were utilized as input, whereas all other variables remained unchanged [24, 27].

### Analysis of population coverage

The IEDB population coverage software, available at http://tools.iedb.org/population/, was used to estimate the expected epitope populations. Predictions were conducted using the inputs, epitopes, and alleles. The default configuration for the IEDB proposed method combines three distinct techniques, which are often used. The most prevalent alleles in humans are readily available for natural selection [26–29].

### Development of a multi-epitope vaccine

The tiny size of epitopes makes them often non-immunogenic when employed as a vaccine [26]. A carrier with potent immuno-stimulatory adjuvants must activate both the innate and adaptive immune systems. The vaccine design's capacity to act as an independent immunogen and produce higher levels of antibodies compared to a single immunogen is mainly achieved via the inclusion of linkers [27]. The LBL, CTL, and HTL epitopes were connected utilizing linkers such as KK, AAY, and GPGPG to enhance the stability and antigenicity of the resulting vaccine. The 'KK' linker was used to join LBL epitopes, the 'AAY' linker was utilized to link CTL epitopes, and the 'GPGPG' linker was employed to connect HTL epitopes. The adjuvant was attached to the vaccination's N-terminal end utilizing the EAAAK linker [29, 35].

### Physical and chemical characteristics of the developed vaccine

The ProtParam service (https://web.expasy.org/protparam/) was used to determine the final vaccine's physiological and biochemical properties, including its peptide composition, hypothetical pI, instabilities index, in-vitro and in-vivo half-life, aliphatic index, molecular weight, and grand mean of hydropathicity.

### Predictions for the secondary structure

The PSIPRED tool (http://bioinf.cs.ucl.ac.uk/) was used to estimate the secondary structure of the vaccine's main amino acid pattern. The chosen analysis from the list of "popular analyses" was "PSIPRED 4.0 (Predict secondary structure)" [28]. The whole sequence was automatically analyzed.

### Tertiary structure prediction and verification

The 3D structure of the planned subunit vaccine candidate was obtained using Robetta, a web server (https://robetta.bakerlab.org/). The system had the target identification and immunization sequence to anticipate the structure. The web interface provided the tertiary structure. The structure was visualized using the Pymol visualization software. Subsequently, the protein structure underwent validation via internet services PROCHECK, ERRAT, and Verify 3D.

## The final vaccine construction using disulfide engineering

The Disulphide by Design (DbD) 2.12 program enhanced stability and was shown with the vaccine structure at http://cptweb.cpt.wayne.edu/DbD2/. A set of criteria (with a chi3 value ranging from 87 to 97 and an energy value of 2.2) was used to choose probable residue pairings. These pairs were then modified to include a cysteine residue [29].

### Prediction of the discontinuous B-cell epitope

The ElliPro server, available at http://tools.iedb.org/ellipro/, was used to predict the non-contiguous B-cell epitopes in the final vaccine structure [30]. The provided input consisted of the PDB structure. The threshold values were maintained at their default defaults. The most miniature residue score, the protrusion index, ranged from 0.5 to 1.0. Similarly, the maximum distance ranged from 4 to 8. The vaccine's 3D structure assigns an ellipsoid score, often referred to as a PI (Protrusion Index) value, to each residue.

### Molecular docking

After analyzing the tertiary structure of the multi-epitope vaccine, TLR-4 (PDB ID: 5IJB) was chosen as the receptor-ligand molecule. The Autodock 4.0 program was used to do docking utilizing the empirical free energy function and the Lamarckian Genetic algorithm [31]. The ligand was assigned fractional charges, and the non-polar hydrogens were connected. The grid map was generated using the Auto grid method, with a grid box size of 90*90*90 and a spacing of 0.35 angstrom. The optimal configuration of the interaction was determined by docking analysis based on the reactive distances and binding energies.

### Modeling of molecular dynamics

Molecular dynamics simulations were conducted utilizing the GROMACS-2019.4 version [32, 33], using the Gromos force field and SPC-2 water model. The energy reduction process removed high-energy configurations, such as bonded terms that were not in equilibrium or steric limitations. The molecules' atoms are then equilibrated by the system utilizing the NVT (constant number of particles, volume, and temperature) ensemble for 500 picoseconds at a temperature of 300 Kelvin. The NPT equilibrium is likewise maintained at a temperature of 300 K and a pressure of 1 bar. Following the equilibration method, a molecular dynamics (MD) simulation lasting 100 ns was conducted for the TLR-4 receptor and the final vaccine design. The trajectory data generated by the MD simulation was analyzed using the GROMACS tools gmx rms, gmx rmsf, gmx gyrate, and gmx hbond to calculate the root-mean-square deviation (RMSD), radius of gyration, and hydrogen bond interactions.

### Codon optimization and in silico cloning of the vaccine

The increased occurrence rates might be ascribed to a specific codon adaptation strategy customized for E. coli K12, the most extensively sequenced prokaryotic bacterium. In the instance of the host species E. coli K12, this approach was used to enhance the creation of the primary sequence of the subunit vaccination protein. Subsequently, the sequence was sent to the JAVA Codon Adaptation Program. The avoidances included the ribosome binding site in prokaryotes, rho-independent transcriptional termination, and the cleavage region of restriction endonucleases. The oligonucleotides of the multi-epitope vaccine were altered and inserted into the pcDNA3.1 ( +) vector at specific restriction sites utilizing the restriction cloning feature of the SnapGene program (GSL Biotech, available at snapgene.com) [34, 35].

## Results

### Diagnostic assessment criteria

Epitopes with cytotoxic or allergic properties should be removed since they might undermine the objective of vaccine development. The antigenicity, allergenicity, toxicity, and immunogenicity of anticipated epitopes were assessed using Vaxigen v2.0, AllerTOP v2.0, Toxinpred, and the IEDB class I immunogenicity program. Epitopes exhibiting elevated immunogenicity and antigenicity, devoid of allergenicity and toxicity, were chosen for further investigation.

## A potential vaccine candidate has been recognized

**Fig. 1:**
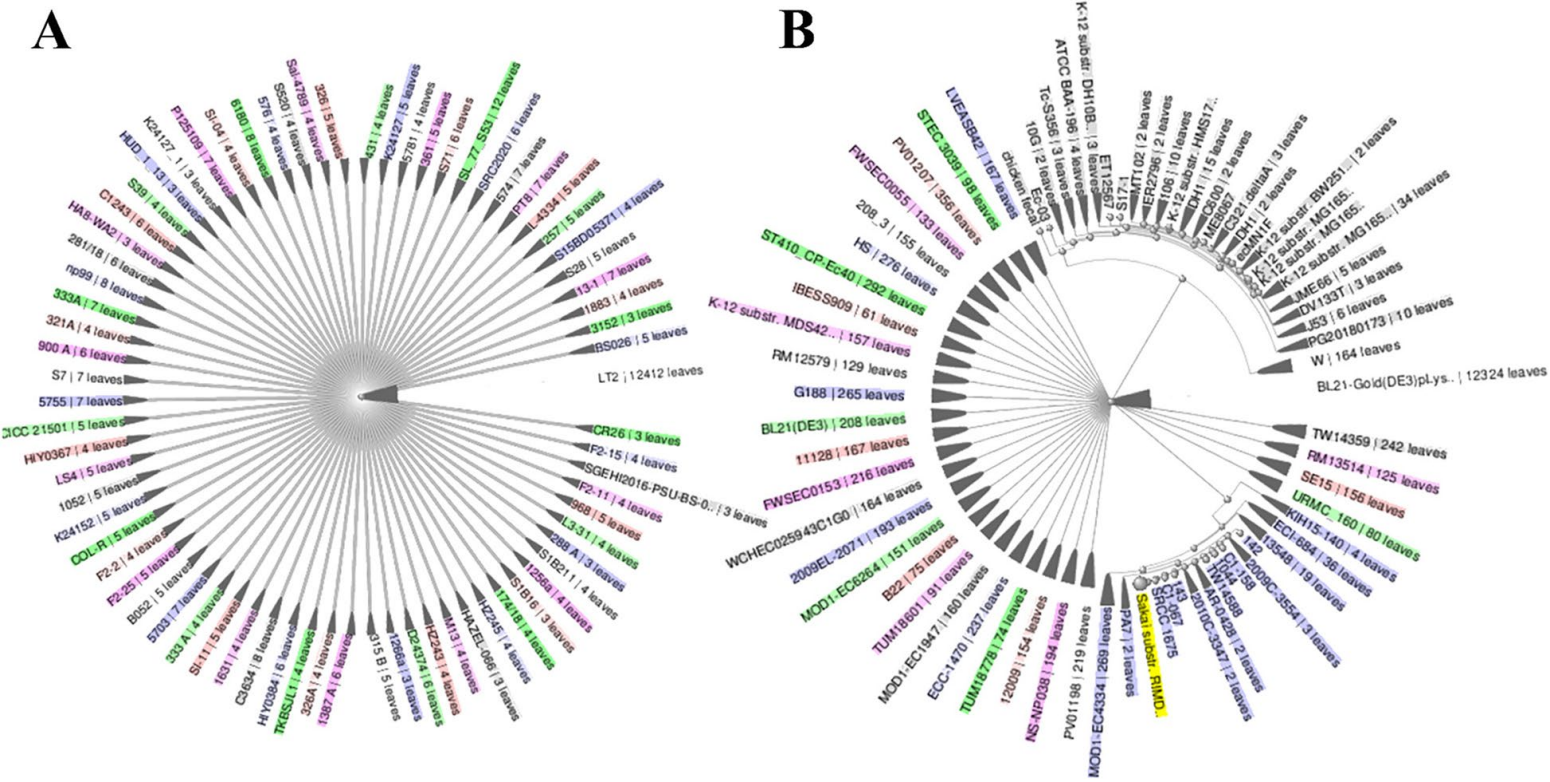
Genome assembly and annotation report from *Salmonella enterica subsp. enterica serovar Typhimurium* str. LT2 and *Escherichia coli* str. K-12 substr. MG1655 strains

This work utilized the NCBI to get 14,114 completely sequenced Salmonella enterica serovar typhimurium genomes and 32,304 fully sequenced E. coli O157:H7 genomes. The complete proteome information for the strain may be accessed at the following URLs: https://www.ncbi.nlm.nih.gov/genome/?term=Salmonella enterica serovar Typhimurium and https://www.ncbi.nlm.nih.gov/genome/?term=E. coliO157:H7. The whole collection of 14,114 genomes for the species S. typhimurium and E. coliO157:H7 may be found at the following website: https://www.ncbi.nlm.nih.gov/genome/browse/#!/prokaryotes/152/ The link provided is https://www.ncbi.nlm.nih.gov/genome/browse/#!/prokaryotes/167/.The user's text is a single period. The proteins encoded by each genome had a median total length of 4.81684 megabases (Mb) for Salmonella enterica serovar Typhimurium and 5.11174 Mb for E. coli O157:H7. Each strain has a different quantity of proteins, with S. typhimurium having a median protein count of 4500 and E. coliO157:H7 having a median protein count of 4726. Figure 1 illustrates the number of genomes discovered in each strain of S. Typhimurium and E. coliO157:H7. The core-pan plot indicates that the pathogen's expected proteome is accessible, and there is a strong probability of incorporating more genes throughout time due to chromosomal flexibility. In addition, examining COG patterns indicated that the core proteins played a significant role in metabolic biogenesis. The distinct assemblage of proteins discovered in Salmonella enterica subsp. enterica serovar Typhimurium str. LT2 and Escherichia coli str. K-12 substrate. MG1655 (reference strain) was associated with the handling and retention of data. The data may be categorized into four groups: RNA processing, replication, transcription and translation, and recombination (Table S1).

The Vaxign dataset found 25 proteins for S. Typhimurium and 15 for E. coli, each with distinct features. Subsequently, 18 S. Typhimurium proteins with potential pathogenicity and nine E. coli proteins with potential pathogenicity were chosen. After evaluating proteins' antigenicity, the number of E. coli and S. typhimurium vaccine candidates was reduced to 5 and 4, respectively. This approach is shown in Table 1. The NCBI webpage was utilized to choose the accession number for proteins once the Uniport database validated their presence. The CELLO algorithm categorized the proteins as cell wall, extracellular, outer membrane, periplasmic, inner membrane, and intracellular proteins. By reducing the probability of the host cell coming into touch with the vaccination, the BLASTp results validated the selectivity of the proteins selected for S. Typhimurium and E. coli. Table 1 presents the results of the first protein screening.

**Table 1:** Screening of *S. typhimurium* and *E. coli* proteins

Bacteria	Name	ACCESSION NUMBER	CELLO analysis		Protein- Sol		VaxiJen
			**LOCALIZATION**	**RELIABILITY**	**SOLpro**	**PI**	
***S. typhimurium***	**MreBCD**	AKH09008.1	Cell wall	3.44	0.41	6.71	0.51
	**LPXTG**	WP_179714467.1	Cell wall	4.12	0.90	4.87	0.68
	**anchor family protein**	APQ79047.1	Cell wall	1.27	0.86	4.13	0.66
	**OMP**	QIG55538.1	OuterMembrane	2.23	0.70	5.90	0.94
***E. coli***	**bamA**	VWQ00961.1	OuterMembrane	2.01	0.50	4.9	0.57
	**flgE**	CAD6011050.1	Extracellular	3.22	0.57	4.41	0.62
	**TonB**	CAD6009997.1	OuterMembrane	3.63	0.81	10.12	0.67
	**ompA**	ACO48717.1	OuterMembrane	4.50	0.56	5.28	0.72
	**ydiY**	CAD6006050.1	OuterMembrane	2.23	0.53	4.96	0.69

### Epitopes for linear B-cells are predicted

We used the ABCpred website to examine the polypeptide's amino acid sequences for the presence of linear epitopes. Table S2 displays 20 predicted ten-mer epitopes for E. coli and 16 predicted ten-mer epitopes for S. typhimurium. According to the specified criteria, only two epitopes (PPPEPVVEPE and GFINNNGPTH) of E. coli and four epitopes (LRQELLLKNS, QPILPKAGDT, ANVYSSVQAR, and VIPALLAAAT) of S. Typhimurium were chosen for the final vaccine formulation (Table 2).

**Table 2:** The multi-epitope vaccine construct was designed using predicted LBL, CTL, and HTL epitopes from pathogens, together with their matching MHC Class I alleles and immunogenic properties

***Bacteria***	***Epitope***		***Allele***	***Formula***	***Antigenicity***	***Toxicity***	***negatively charged residues (Asp* + *Glu)***	***positively charged residues*** ***(Arg* + *Lys)***	***Mol wt***	***pI***	***GRAVY***	***Aliphatic index***
***S. typhimurium***	**B-cell**	LRQELLLKNS	–-	C_53_H_96_N_16_O_16_	0.14	Non-toxin	1	2	1213.44	8.75	-0.450	156
		QPILPKAGDT	–-	C_46_H_78_N_12_O_15_	0.59	Non-toxin	1	1	1039.20	5.84	-0.510	88
		ANVYSSVQAR	–-	C_46_H_75_N_15_O_16_	0.95	Non-toxin	0	1	1094.19	8.79	-0.240	78
		VIPALLAAAT	–-	C_44_H_78_N_10_O_12_	0.50	Non-toxin	0	0	939.16	5.49	2.120	186
	**CTL**	RPSQRHGSKY	HLA-A*01:01	C_51_H_82_N_20_O_15_	1.33	Non-toxin	0	3	1215.34	11.0	-2.450	0.0
		TQDENPVVHF	HLA-A*02:01	C_52_H_76_N_14_O_18_	0.33	Non-toxin	0	2	1185.26	4.35	-0.830	58
		NMYKDSHHPA	HLA-A*02:01	C_51_H_74_N_16_O_16_S_1_	0.16	Non-toxin	1	1	1199.31	6.92	-1.730	10
		TMDHARHGFL	HLA-A*02:01	C_51_H_77_N_17_O_14_S_1_	0.04	Non-toxin	1	1	1184.34	6.61	-0.520	49
	**HTL**	SLQIRLILAVLVALG	HLA-DRB1*01:01	C_74_H_135_N_19_O_18_	1.02	Non-toxin	0	1	1579.00	9.47	2.053	234
		ARYIATAVPVKS	HLA-DPA1*02:01/DPB1*14:01	C_58_H_98_N_16_O_16_	0.41	Non-toxin	0	2	1275.51	9.99	0.458	105
		KILAAVIPALLA	HLA-DPA1*02:01/DPB1*14:01	C_58_H_105_N_13_O_13_	0.71	Non-toxin	0	1	1192.55	8.75	2.192	220
		FYFISNGPRELL	HLA-DRB3*02:02	C_70_H_102_N_16_O_18_	0.09	Non-toxin	1	1	1455.68	6.0	0.175	97
***E. coli***	**B-cell**	SVGKYSASVK	–-	C_45_H_76_N_12_O_15_	0.81	Non-toxin	0	2	1025.17	9.70	-0.170	68
		FRLVDSNGSV	–-	C_47_H_76_N_14_O_16_	1.40	Non-toxin	1	1	1093.20	5.84	0.150	97
		PPPEPVVEPE	–-	C_50_H_76_N_10_O_17_	0.49	Non-toxin	3	0	1089.21	3.67	-1.010	58
		GFINNNGPTH	–-	C_46_H_67_N_15_O_15_	-0.24	Non-toxin	0	0	1070.13	6.74	-0.950	39
		WSLWGNASNT	–-	C_51_H_70_N_14_O_16_	0.90	Non-toxin	0	0	1135.20	5.52	-0.590	49
	**CTL**	KLVFQEGVSA	HLA-A*02:01	C_49_H_80_N_12_O_15_	0.55	Non-toxin	1	1	1077.25	6.0	0.470	107
		SIPNTLMAAK	HLA-A*03:01	C_45_H_80_N_12_O_14_S_1_	0.17	Non-toxin	0	1	1045.26	8.47	0.330	98
		GLLYTSVHQV	HLA-A*02:01	C_51_H_81_N_13_O_15_	-0.23	Non-toxin	0	0	1116.28	6.74	0.610	136
		WRADTKSNVY	HLA-B*27:05	C_55_H_82_N_16_O_17_	0.34	Non-toxin	1	2	1239.35	8.59	-1.310	39
		HTDRRTTLSL	HLA-B*07:02	C_49_H_86_N_18_O_17_	0.68	Non-toxin	1	2	1199.33	9.61	-1.100	78
	**HTL**	LVFSYAQPFKKY	HLA-DRB5*01:01	C_75_H_107_N_15_O_17_	0.018	Non-toxin	0	2	1490.77	9.53	-0.075	65
		NGFFRLVDSNGS	HLA-DRB1*04:05	C_57_H_85_N_17_O_19_	0.69	Non-toxin	1	1	1312.40	5.84	-0.317	56
		ILFKINGTTEIQ	HLA-DRB3*02:02	C_63_H_105_N_15_O_19_	0.71	Non-toxin	1	1	1376.62	6.0	0.325	130
		SPVFAGGVEYAI	HLA-DQA1*05:01/DQB1*03:01	C_57_H_84_N_12_O_17_	0.61	Non-toxin	1	0	1209.36	4.0	0.942	97
		MKLLKTVPAIVM	HLA-DRB1*01:01	C_62_H_114_N_14_O_14_S_2_	0.49	Non-toxin	0	2	1343.79	10	1.333	154

### Epitope prediction for cytotoxic T cells

The NetMHCpan 4.1 dataset was employed to forecast MHC Class I interaction sequences (Additional file: Table S3). The most prominent sequences from E. coli (KLVFQEGVSA and HTDRRTTLSL) and S. Typhimurium (RPSQRHGSKY, NMYKDSHHPA, and TMDHARHGFL) with significant antigenicity and positive immunity index were selected and listed in Table 2.

### Epitope prediction for helper T cells

The IEDB website's MHC class II binding peptide identification technique was employed to forecast sequences associated with MHC class II molecules. The IEDB-recommended approach was employed for MHC class II binding analysis, and a reference group consisting of 27 alleles was used. The protein sequence was analyzed, and four peptides from S. Typhimurium (SLQIRLILAVLVALG, ARYIATAVPVKS, KILAAVIPALLA, and FYFISNGPRELL) and five HTL epitopes from E. coli (LVFSYAQPFKKY, NGFFRLVDSNGS, ILFKINGTTEIQ, SPVFAGGVEYAI, and MKLLKTVPAIVM) were discovered. This information can be seen in the Additional file (Table S4). The protein-based multi-epitope vaccine was created using the final epitopes selected from Table 2. Table 2 indicates immunogenic epitopes with suitable features were chosen from the bacteria E. coli and S. typhimurium.

### Population coverage analysis of the selected epitopes

**Fig. 2:**
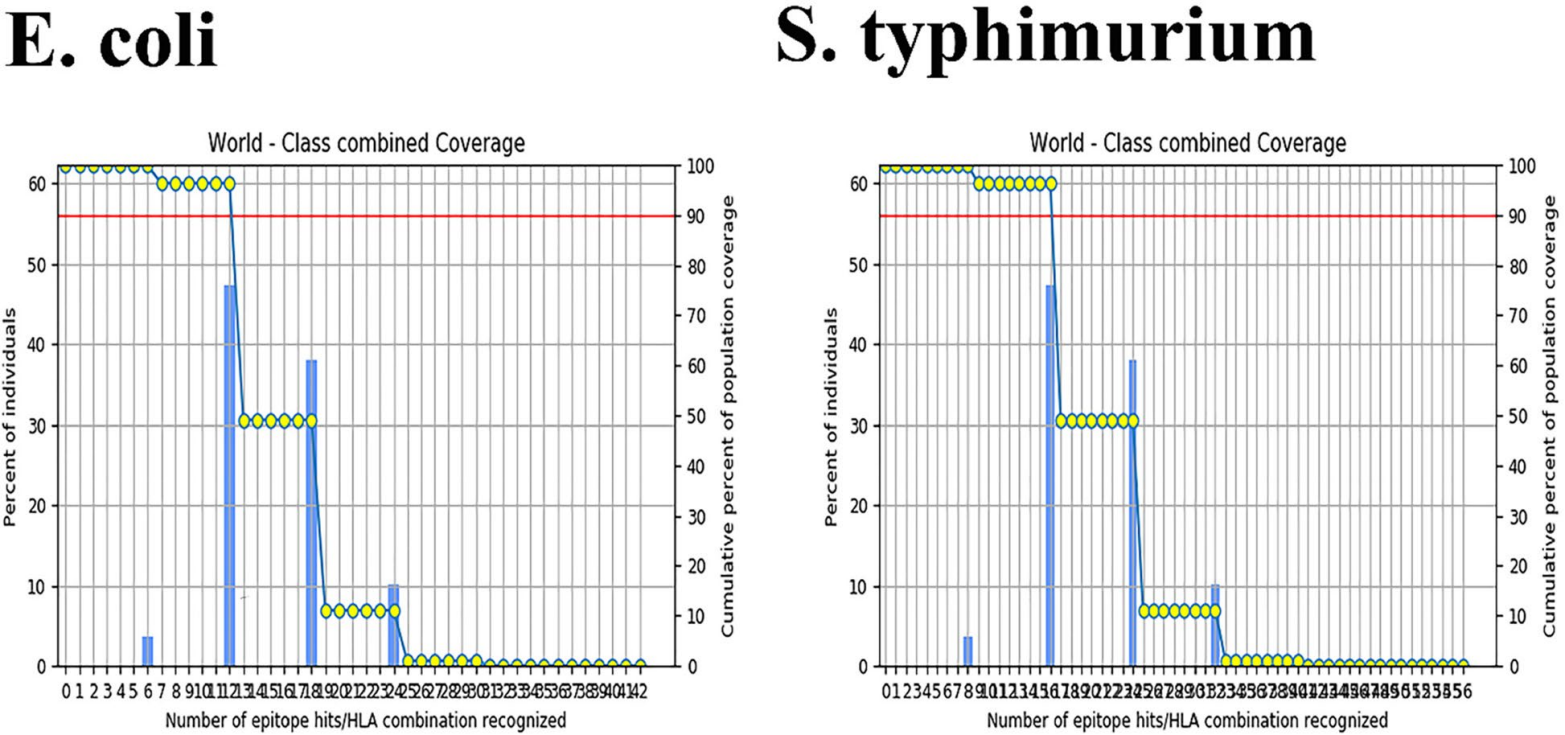
Population Coverage Calculation Result for the 6 chosen epitopes of *S. typhimurium* and the 8 chosen epitopes of E. coli. The overlap of selected epitopes with human alleles for *S. typhimurium* and *E. coli* bacterium was found to be 99.94%

The population coverage for the three selected B-cell epitopes (QPILPKAGDT, ANVYSSVQAR, and VIPALLAAAT) and three selected T-cell epitopes (CTL = RPSQRHGSKY, HTL = SLQIRLILAVLVALG, and KILAAVIPALLA) (1 CTL and 2 HTL) for S. Typhimurium, as well as the selected B-cell epitope (LRQELLLKNS) and T-cell epitopes (CTL = KLVFQEGVSA and HTDRRTTLSL, HTL = SPVFAGGVEYAI) for E. coli, were calculated for their binding HLA alleles, as depicted in Fig. 2. The study discovered that the selected epitopes of the Salmonella Typhimurium bacterium have a 99.94% overlap with human alleles, specifically HLA-A*01:01, HLA-A*03:01, HLA-B*27:01, HLA-B*27:02, HLA-B*27:03, HLA-B*27:04, HLA-B*27:05, HLA-B*27:06, HLA-B*27:07, HLA-B*27:08, HLA-DMA*01:01, HLA-DMA*01:01, HLA-DMB*01:01, and HLA-DQB1*02:01. The study found that the selected epitopes for the E.coli bacterium have a 99.94% overlap with human alleles, specifically HLA-A*01:01, HLA-A*03:01, HLA-B*27:01, HLA-B*27:02, HLA-B*27:03, HLA-B*27:04, HLA-B*27:05, HLA-B*27:06, HLA-B*27:07, HLA-B*27:08, HLA-DMA*01:01, HLA-DMA*01:01, HLA-DMB*01:01, and HLA-DQB1*02:01. Additionally, the average hit was 20.6.

### The multi-epitope vaccine candidate's development

**Fig. 3:**
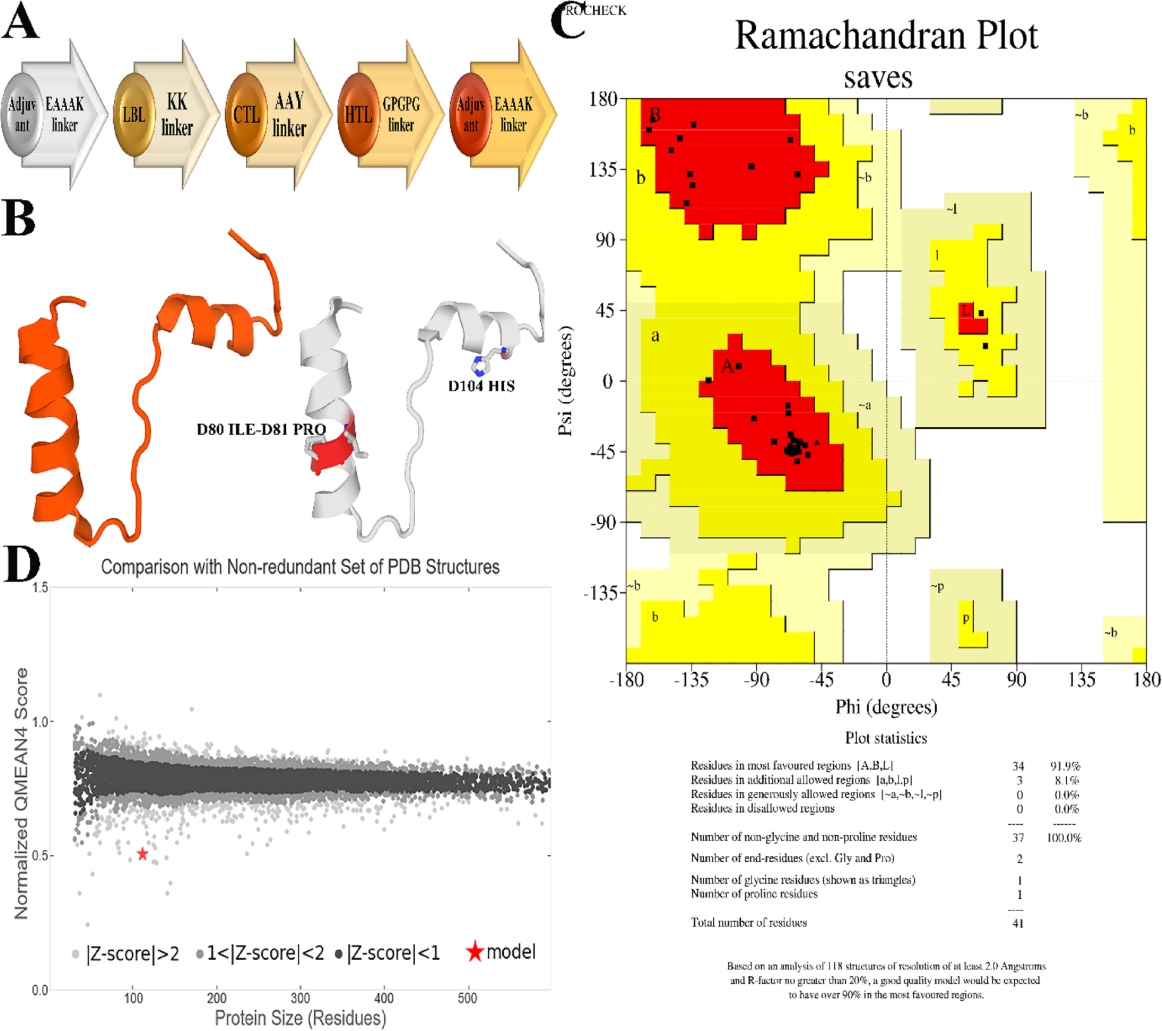
**A** The final multi-epitope vaccine construct's structural organization of B and T-cell epitopes, linkers, and adjuvant. **B** 3D models of vaccine construct. **C** Ramachandran Plot for vaccine. MolProbity Results of Ramachandran plot show that MolProbity Score, Ramachandran Favoured, Clash Score and Ramachandran Outliers were 0.90, 100.00%, 1.54 and 0.00%. **D** Coordinates with QMEAN local scores in the B-factor column

Consequently, a multi-peptide vaccine was created by combining many epitopes, such as LBL, CTL, and HTL, using suitable linkers at crucial positions. The final vaccination was created by combining three linkers, expressly specified as KK, AAY, and GPGPG. The AAY and GPGPG linkers were included in the intra-epitope region to connect the CTL and HTL epitopes. Subsequently, the molecular adjuvant (MSPSVRHSPSVRH) was introduced at both the N-terminal and C-terminal ends of the selected vaccine sequence. This enhanced the efficiency of the immune response. Figure 3A depicts the arrangement of adjuvant and epitopes and their connecting linkers.

### Solubility, physicochemical characteristics, and prediction of secondary structure

Table 3 presents the established vaccine composition. The ProtParam website provided data indicating that the aliphatic index, GRAVY, theoretical PI, instability rating, and molecular weights of the multi-epitope vaccine were found to be 83.01, -0.273, 10.31, 57.86, and 25,344.41 Da, respectively. The molecular mass of the polypeptide was determined, and this was the most essential element of the phase [36–38]. Proteins with a molecular weight of less than 110 kDa are more suitable for isolation and efficient use in vaccine development due to their simplicity. Based on the study on physicochemical qualities, it was anticipated that the vaccine would possess excellent solubility, stability, indispensability, hydrophilicity, thermostability, and stability even after overexpression in E. coli.

**Table 3:** The physicochemical properties of the generated sequence of vaccine structures designed in this study

Epitope composition	group	Vaccine sequence	aliphatic index	GRAVY	PI	instability index	Molecular mass (Da)	VaxiJen	homology
Multi epitope vaccine	Adjuvant + CTL + HTL + Adjuvant	MSPSVRHSPSVRHEAAAKPPPEPVVEPEKKGFINNNGPTHKKLRQELLLKNSKKQPILPKAGDTKKANVYSSVQARKKVIPALLAAATKKKLVFQEGVSAAAYHTDRRTTLSLAAYRPSQRHGSKYAAYNMYKDSHHPAAAYTMDHARHGFLAAYSLQIRLILAVLVALGGPGPGARYIATAVPVKSGPGPGKILAAVIPALLAGPGPGSPVFAGGVEYAIEAAAKMSPSVRHSPSVRH	83.01	-0.273	10.31	57.86	25,344.41	**0.63**	non-homologue

Furthermore, the vaccine had a half-life of 30 h in human reticulocytes, more than 20 h in yeast, and more than 10 h in E. coli (in vivo). Table 3 summarizes the findings from the analysis of the physicochemical parameters. According to the results obtained from the PSIPRED server, it has been determined that random coils are formed by a combination of just 47 amino acids, α-helixes are formed by a combination of 99 amino acids, and a combination of 26 amino acids forms β-strands. The vaccine's secondary structure projection indicates that 57.55% consists of random coils, 15.11% forms α-helixes, and 27.32% consists of β-strands.

### Tertiary structure identification and vaccine construct verification

The Galaxyrefine server predicted five tertiary structures for synthesizing the vaccine. These configurations were further analyzed using a Ramachandran plot to choose the optimal model (Table 4). The selection of Model 3 was based on its optimal arrangement of residues within allowed regions, as shown in Fig. 3B. The Ramachandran plot indicated that most residues were found in the preferred conformation (100%), with no outliers in the Ramachandran or Rotamer regions (0.00% for both). The distribution of residues is as follows: 91.9% in the most favoured areas, 8.1% in extra authorized regions, and 0.00% in liberally allowed or banned locations (Fig. 3C).

**Table 4:** Models after refinement using Galaxyrefine server

Model	GDT-HA	RMSD	MolProbity	Clash score	Poor rotamers	Rama favored
**Initial**	1.0000	0.000	1.099	3.1	0.0	100.0
**MODEL 1**	0.9512	0.450	1.465	3.0	3.1	100.0
**MODEL 2**	0.9634	0.440	1.266	1.5	3.1	100.0
MODEL 3	0.9390	0.422	1.089	3.0	0.0	100.0
**MODEL 4**	0.9512	0.439	1.465	3.0	3.1	100.0
**MODEL 5**	0.9329	0.456	0.890	1.5	0.0	100.0

**Fig.4:**
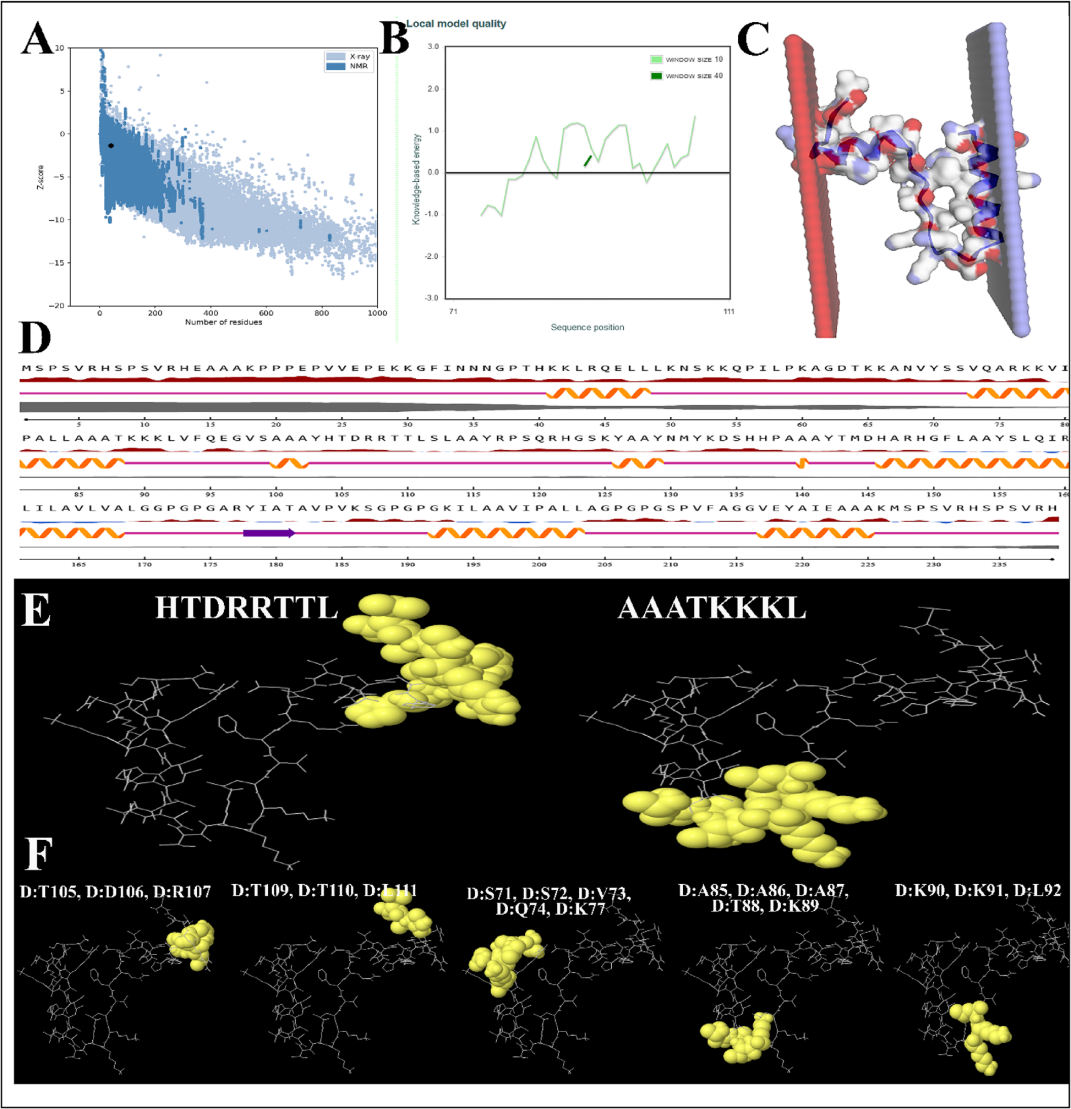
**A** The predicted structure was validated using ProSA with a Z score of—1.34. **C** Energy quality of the local Vaccine model showed a good B-factor. **C** Cross-sectional image of the vaccine structure. **D** The second structure of the vaccine structure. Explanation of the symptoms is shown below. **E** The 3D representations of predicted Linear B-Cell Epitope. **F** The 3D representations of structural proteins

This structure is thermodynamically stable, with a quality factor of 80.7692 (ERRAT Complete) and a Verify-3D score of 95.93, respectively. Figure 4C illustrates the Ramachandran plot of the structure of the multi-epitope protein. Figure 3D illustrates the comparison using a non-redundant collection of PDB structures.

### Vaccine construct modeling, refining, and validation

The Z-score of the models was selected for enhancement from the top 5 predicted models (Table 4). The enhanced model's overall and specific performance was assessed using the ProSA-web tool, resulting in a Z-score of -1.34. The Z-score of this protein is within the measured range for natural proteins of comparable size, as shown in Figs. 4A and B. Figures 4C and D provide a graphical depiction of 239 predictions for residues in the vaccination sequence.

### Disulfide engineering to stabilize vaccine protein

Table 5 shows the potential residue pairings that might create a disulfide bond after the vaccine sequence underwent processing using the Disulfide by Design 2.13 tool. No disulfide bonds were chosen. Only one residue pair was excluded from selection owing to its deviation from the typical limit of bond energy (less than 2.2 kcal/mol) and three angles (between + 87° and + 97°).

**Table 5:** Disulfide engineering of the final multiepitope vaccine

Res1 Chain	Res1 Seq #	Res1 AA	Res2 Chain	Res2 Seq #	Res2 AA	Chi3	Energy	Sum B-Factors
D	107	ARG	D	110	THR	+ 123.15	6.29	0

### Mapping of discontinuous B‑cell epitopes in the vaccine protein

B cells produce antibodies and cytokines to counteract foreign antigens, strengthening humoral immunity [40, 41]. Thus, the protein domain must possess a enough quantity of B-cell epitopes. Utilizing the ElliPro tool on the IEBD server, an analysis was conducted to determine the presence of conformational B-cell epitopes. The analysis identified three non-adjacent (discontinuous) epitopes and two adjacent (linear) epitopes. The ElliPro server identified a total of 2 Linear B-cell epitopes with ratios of 0.707 (HTDRRTTL) and 0.643 (AAATKKKL), as well as 5 Discontinuous Epitopes with ratios ranging from 0.602 to 0.732. These epitopes have 19 residues and are shown in Figs. 4E and F. This information is summarized in Tables 6 and 7.

**Table 6:** Predicted linear epitope(s)

No	Chain	Start	End	Peptide	Number of residues	Score	3D structure
1	D	104	111	HTDRRTTL	8	0.707	Figure 4E
2	D	85	92	AAATKKKL	8	0.643	Figure 4E

**Table 7:** Predicted discontinuous epitope(s)

No	Residues	Number of residues	Score	3D structure
1	D:T105, D:D106, D:R107	3	0.732	Figure 4F
2	D:T109, D:T110, D:L111	3	0.691	Figure 4F
3	D:S71, D:S72, D:V73, D:Q74, D:K77	5	0.678	Figure 4F
4	D:A85, D:A86, D:A87, D:T88, D:K89	5	0.668	Figure 4F
5	D:K90, D:K91, D:L92	3	0.602	Figure 4F

### Molecular docking of vaccine with TLR4 receptor

**Fig. 5:**
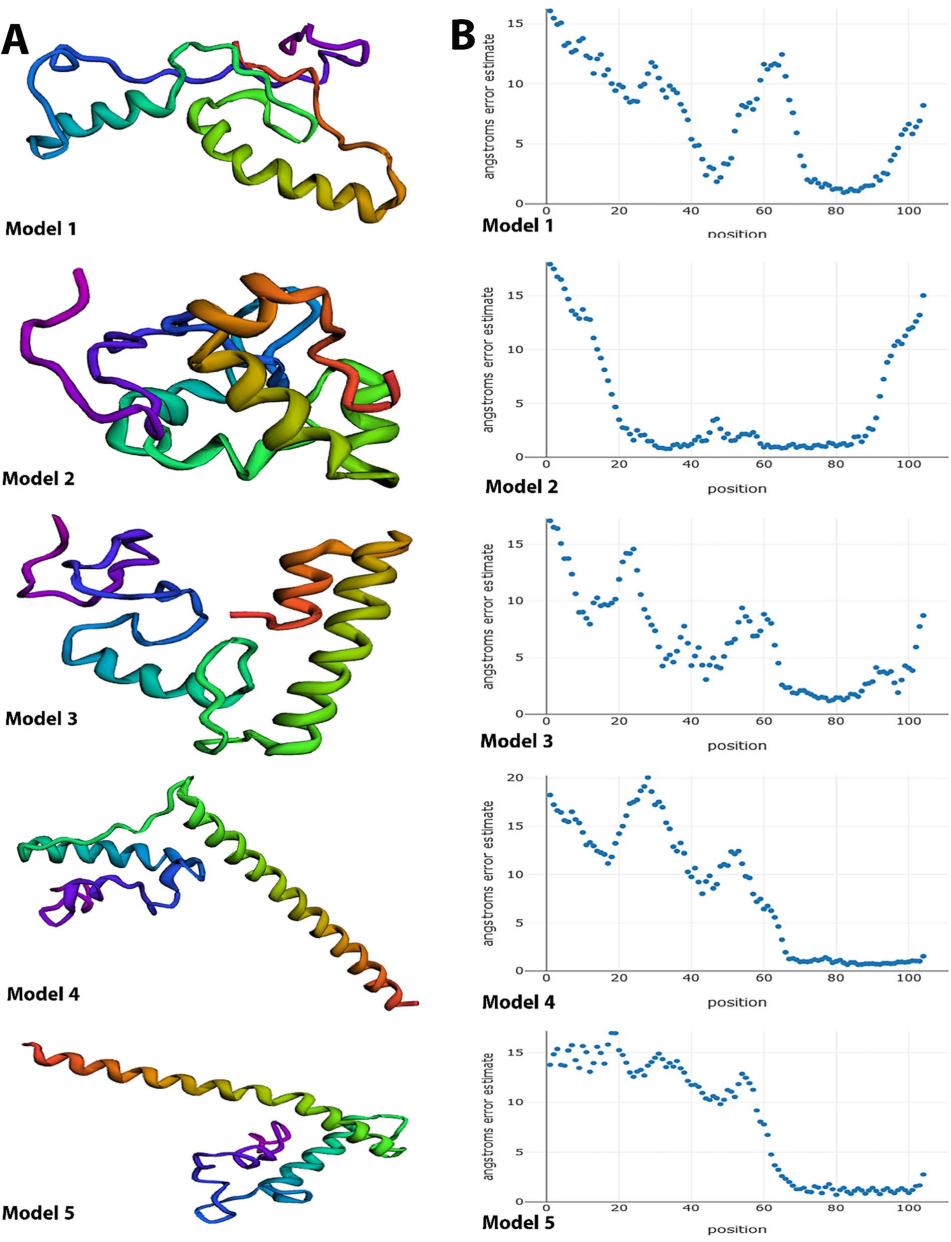
**A** 3D structure of the various models predicted for the vaccine by the Robetta site. **B** Angstroms error estimated of various vaccine models

Four distinct models of the vaccine structure were analyzed, and model 1 was chosen as the optimal model based on the data acquired from the Robetta site. The residue in this model achieved the maximum energy level of -16.194 kcal/mol. The alignment cluster 1 for the selected vaccination model may be found in Table S6 of supplemental materials 1. Furthermore, the anticipated models were shown in Fig. 5.

**Fig. 6:**
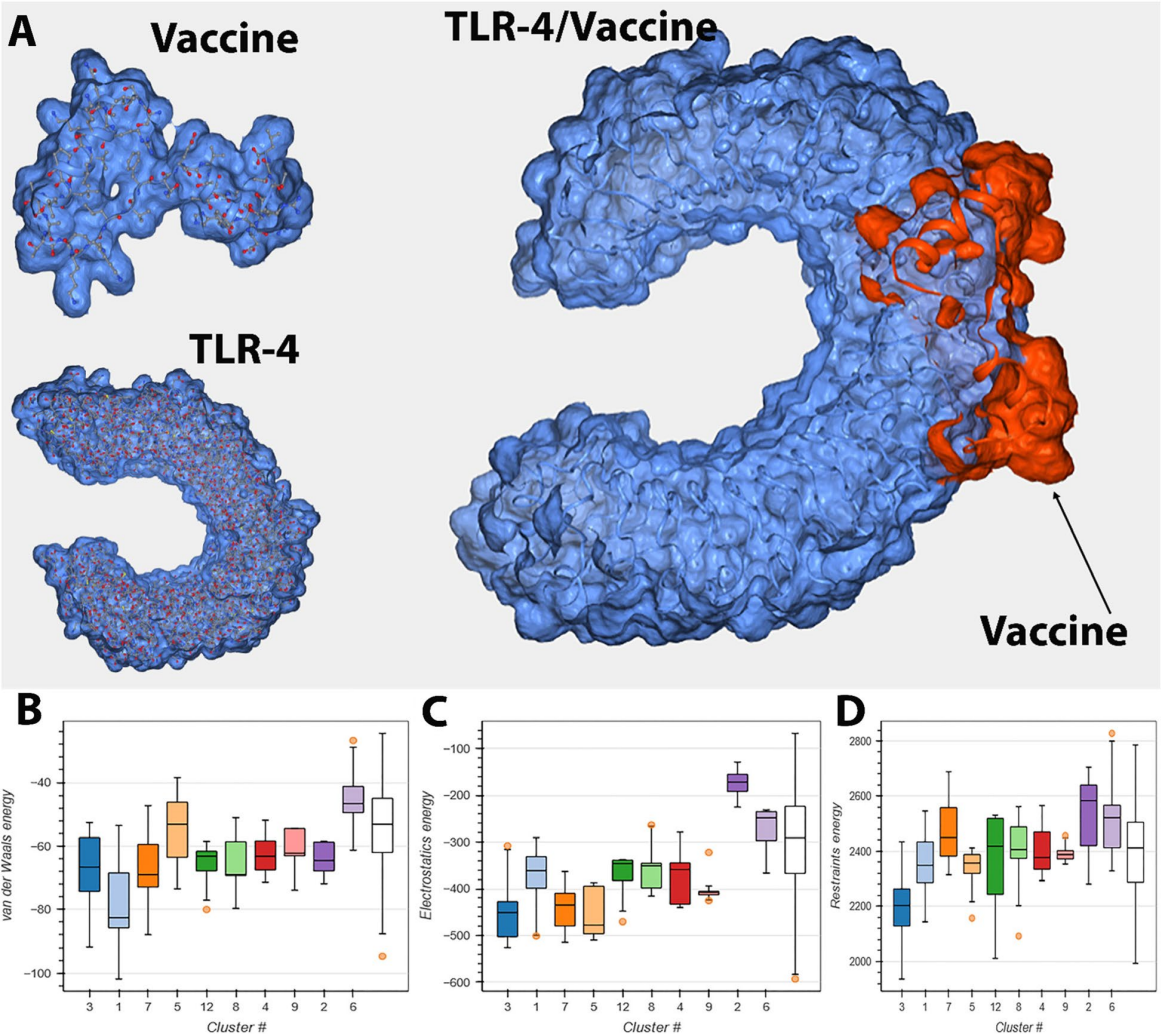
**A** The vaccine protein underwent docking analysis with TLR4 to determine the binding affinity between the vaccine construct and this receptor. **B** Van der Waals energy from TLR-4/Vaccine docking, **C** Electrostatic energy from TLR-4/Vaccine docking, **D** Restraints violation energy from TLR-4/Vaccine docking

The vaccination protein was subjected to docking studies with TLR4 to assess the binding affinity between the vaccine construct and this receptor. The grid box placed in the active site area was used for conducting molecular docking (Fig. 6A). The third cluster was chosen as the optimal cluster. The Van der Waals energy was measured to be -77.0 ± 8.8, whereas the Electrostatic energy was found to be -446.1 ± 77.0. The TLR4 receptor and the vaccine candidate were observed using Pymol. The analysis yielded ten distinct clusters, as shown in Table 8. These clusters were shown in Figs. 6B, C, and D, representing Van der Waals energy, Electrostatic energy, and Restraints violation energy, respectively.

**Table 8:** Complete docking results of the top 10 clusters obtained from the HADDOCK site

HADDOCK	Cluster 3	Cluster 1	Cluster 7	Cluster 5	Cluster 12	Cluster 8	Cluster 4	Cluster 9	Cluster 2	Cluster 6
HADDOCK score	59.9 ± 13.0	72.6 ± 8.9	87.3 ± 11.5	104.9 ± 14.9	106.5 ± 35.7	107.9 ± 20.2	115.5 ± 11.1	121.7 ± 2.9	134.9 ± 6.8	148.9 ± 16.8
Cluster size	10	25	6	8	4	5	9	5	13	6
RMSD from the overall lowest-energy structure	0.9 ± 0.6	2.3 ± 0.5	5.0 ± 0.1	7.0 ± 0.0	1.6 ± 0.2	6.4 ± 0.1	2.5 ± 0.1	2.6 ± 0.1	6.1 ± 0.1	3.6 ± 0.1
Van der Waals energy	-77.0 ± 8.8	-80.8 ± 16.2	-66.3 ± 15.4	-60.5 ± 8.8	-66.2 ± 8.2	-69.2 ± 7.5	-62.9 ± 6.7	-63.4 ± 6.9	-66.7 ± 5.0	-48.6 ± 7.9
Electrostatic energy	-446.1 ± 77.0	-431.4 ± 50.6	-447.6 ± 41.6	-471.7 ± 49.5	-375.2 ± 55.4	-355.7 ± 59.2	-396.0 ± 44.6	-390.1 ± 40.0	-190.3 ± 34.0	-288.2 ± 54.6
Desolvation energy	15.0 ± 6.7	21.6 ± 4.0	3.4 ± 3.1	30.8 ± 3.8	13.3 ± 5.1	14.2 ± 3.0	21.7 ± 3.1	25.2 ± 2.2	-0.6 ± 3.8	10.8 ± 3.6
Restraints violation energy	2111.4 ± 126.9	2181.0 ± 60.8	2397.8 ± 67.5	2289.9 ± 87.7	2344.8 ± 209.7	2340.2 ± 149.0	2359.9 ± 72.7	2378.9 ± 18.3	2403.1 ± 81.7	2443.8 ± 92.9
Buried Surface Area	2690.8 ± 79.2	2785.2 ± 72.8	2509.0 ± 59.6	2483.2 ± 114.9	2617.7 ± 125.8	2459.4 ± 113.1	2290.3 ± 64.3	2365.8 ± 24.5	2241.7 ± 43.0	2169.8 ± 53.5
Z-Score	-1.8	-1.3	-0.7	-0.0	0.0	0.1	0.4	0.6	1.1	1.7

### Molecular dynamic simulation

**Fig. 7:**
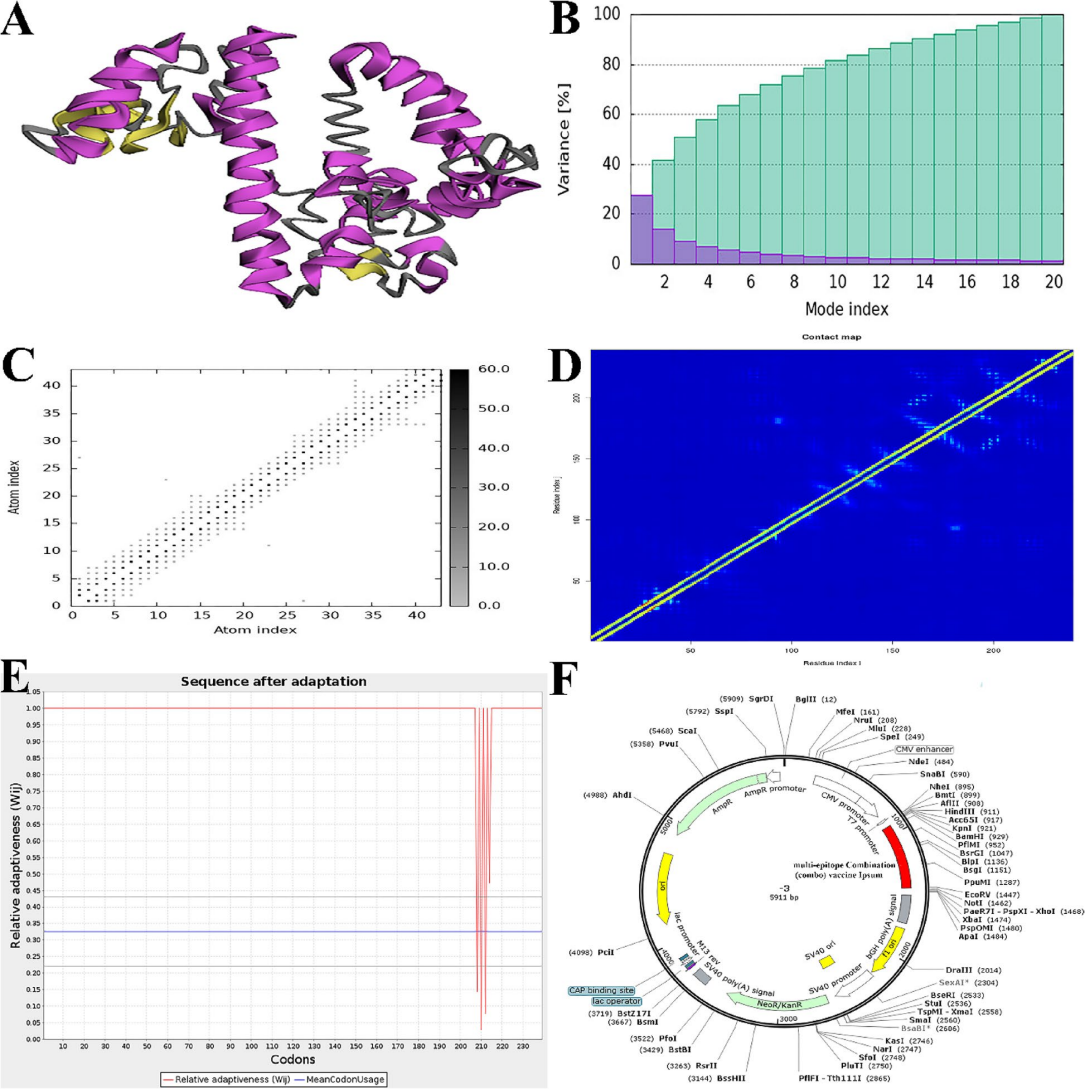
**A** NMA mobility in protein structure, variance **(B),** covariance **(C)** and complex elastic network covariance **(D)** of molecular dynamics simulation of a multi-epitope vaccination that is bound to the TLR-4 receptor. **E** The process of optimizing codons in vaccine constructions targeting *E. coli* K12 strain. **F** Vaccine cloning using computational methods. The red region inside the pcDNA3.1( +) expression vector represents the multi-epitope vaccination insert

The molecular dynamics simulation was performed using iModS. IModS examines the structure by altering a complex force field at various intervals. The heat map exhibits a substantial correlation region and a small root mean square deviation (RMSD), indicating robust relationships between individual residues. Figure 7 offers a thorough explanation of the IModS results. Figure 7A illustrates the NMA mobility in protein structure. Figures 7B illustrate the variability, 7C shows the interdependence, and 7D display the intricate elastic network.

### Adaptation of codons and in silico cloning

Codon adaptation is the optimal approach for enhancing translational efficiency due to the constraints imposed by variations in codon use, which might impede the translation of foreign genes. We improved the codon use of our developed vaccine for the E. coli K12 strain by using the JCat tool. The optimized sequence exhibited a codon adaptation index (CAI) of 0.963, suggesting that it may be efficiently expressed in the E. coli host, which has a GC content of 53.55% (Fig. 7E). The results indicated that the prokaryotic ribosome binding site, regions susceptible to cleavage by restriction enzymes, and rho-independent transcription terminators were deleted due to adaptation. The altered sequence of codons from the vaccine design was introduced into the E. coli expression vector pcDNA3.1( +) at the EcoRI and BamHI restriction sites, as seen in Fig. 7F. In order to facilitate the immune-chromatographic purification of the recombinant vaccine, a 6-histidine tag was further included. The clone had a length of 5911 base pairs.

## Discussion

*Vaccination* is a conventional technique used to enhance the host's immune system against a specific infection [39, 40]. Different types of vaccinations, such as live or attenuated ones, are expensive, need a significant amount of time, and have a lengthy process before they can be made available for sale [41, 42]. The attenuated vaccine's substantial antigenic load results in suboptimal passive immunization and allergic responses [43]. The advancement of multi-omics technology has facilitated the identification of epitopes that elicit a robust immune response. Immunoinformatic approaches have been used to develop vaccines against M. leprae and N. meningitidis driven by several epitopes [44, 45]. An emerging and concerning bacteria is the pathogen responsible for producing intestinal diseases. Our current understanding of the host's immune response to invasive pathogen infections causing intestinal illness has made limited advancements. Recognition of pathogen-associated molecular patterns is crucial for the functioning of the innate immune defence system. Pattern-associated molecular patterns (PAMPs) are detected by pattern recognition receptors (PRRs), including Toll-like receptors and components of the complement system. By detecting the presence of peptidoglycan and lipoteichoic acid, it has been shown that Toll-like receptor 2 (TLR2) plays a crucial role in the innate immune response to bacteria that are responsible for causing intestinal disease.

Furthermore, apart from the direct interaction between the microbe and the phagocyte, there is also an indirect pathway facilitated by opsonins, which are made up of antibody and complement subunits. In response to the activation of the alternative complement pathway, the complement protein is deposited on the surface of the bacterial cell. Phagocytes that detect this protein then react appropriately [46]. The dissemination of pathogenic bacteria that cause intestinal diseases to other anatomical regions may be attributed to the immune system's failure to eliminate them. Hence, the development of possible vaccinations might be advantageous in combating the germs responsible for gut diseases.

In the current study, a possible vaccine is the most effective approach for avoiding infection by disease-causing bacteria in the intestines. Given that more antigenicity leads to a more robust immune response, we chose six proteins associated with the pathogenicity of the bacteria. Various databases were used to anticipate potential T and B-cell epitopes, and their suitability as vaccination candidates was assessed. HTL epitopes are essential for eliciting both humoral and cell-mediated immune responses. In contrast, CTL epitopes are crucial in recognizing foreign antigen components on MHC-I molecules and eliminating target cells. B-cell epitopes play a vital role in the development of epitopic vaccines. Therefore, it is crucial to predict these epitopes in order to produce an immunotherapeutic and prophylactic vaccination. Predicting an organism's capacity to generate antigenic sequence data is essential as it determines the likelihood of the microorganism's proteome being recognized by immune cells in the human body. Geographical variations influence vaccine development since they worldwide impact the activation of HLA alleles, resulting in an altered variety [47, 48].

The T-cell epitopes (CTL and HTL) that were finished were found to encompass 99.94% of the population in terms of the HLA alleles they are connected with. Finally, to provide an effective and dependable vaccine design, we included KK, AAY, and GPGPG linkers between the LBL, CTL, and HTL epitopes to ensure a robust immune response. An adjuvant called (MSPSVRHSPSVRH) was used to enhance the immunological response. It was connected to the N-terminal and C-terminal of the vaccination using an EAAAK linker [49, 51].

EAAAK is a peptide linker that forms a tight alpha-helix structure, with intramolecular hydrogen bonding that results in a densely packed backbone. EAAAK linkers facilitate the practical separation of functional domains by maintaining a consistent distance, minimizing interaction across epitopes, and preserving their distinct functional features. This facilitates the efficient segregation of domains in a bifunctional fusion protein [50–53].

It was proven that GPGPG linkers can elicit TH lymphocyte (HTL) responses, which are essential for a multi-epitope vaccination. Additionally, the GPGPG linker is a helpful tool for disrupting junctional immunogenicity and restoring the immunogenicity of individual epitopes.

The AAY (Ala-Ala-Tyr) linker is the location where proteasomes in mammalian cells break down proteins. Consequently, when epitopes are connected via an AAY linker, they are efficiently segregated inside the cells, decreasing junctional immunogenicity. The AAY linker also enhances the immunogenicity of the multi-epitope vaccination [53–55].

The resulting vaccine has the optimal molecular mass for chimeric vaccines, indicating its inherent stability, hydrophilicity, and thermostability. The vaccine's ability to stimulate an immune response and lack of allergenic properties indicated its efficacy as a vaccination.

The water content in vaccine manufacture is crucial as it facilitates the rapid separation and purification of the recombinant protein from its E. coli host. The vaccination protein exhibited solubility when produced at high levels in the E. coli host. The proposed chimeric vaccine had a satisfactory stability index, indicating its potential as a candidate for vaccination. The brief duration of epitopes is often a matter of concern during the development of vaccinations. The vaccine constructions exhibited promising structural quality, as shown by the Z-score and Verify3D. Based on the validation results, the tertiary structure is deemed suitable for future investigation. Prote stability is paramount in several biological and therapeutic contexts [49–52]. The protein's thermostability was improved using disulfide engineering techniques and introducing two specific mutations. E. coli cell culture technology is often used for the large-scale production of recombinant proteins [50–53].

Codon modification was performed on E. coli strain K12 to achieve practical expression in the host. The molecular docking analysis demonstrated that the proposed vaccine binds with the TLR4 receptor-binding area with low binding energy, suggesting a strong binding affinity. A molecular dynamics simulation of the docked complex was used to assess the mobility and stability of the TLR4-vaccine complex in the biological system [54, 55]. The graph also illustrated the exceptional stability of the protein–protein combination, with each residue exhibiting a decreased deformation. In summary, molecular dynamics simulations demonstrated that the suggested vaccine exhibits negligible molecular deformability and is sufficiently stable. In addition, the results of the immunological simulation demonstrated that the vaccine's design elicited an immune response against bacteria responsible for intestinal illness, like the immune response seen in viral infections. Following vaccination, it was shown that the secondary and tertiary defensive mechanisms exhibited greater strength compared to the initial immunological responses. This was evident via the substantial synthesis of antibodies and the effective removal of antigens. Immunization has been seen to enhance both cell-mediated and humoral immunity, as evidenced by an increase in B-cells (memory B-cell and plasma B-cell) and T-cells (cytotoxic and helper T-cells). The heightened abundance of antigen-processing cells, such as dendritic cells and macrophages, provides evidence that the vaccine's structure can stimulate adequate antigen processing and presentation to CD4 + and CD8 + cells. Additional in vitro and in vivo experimental testing is necessary to validate the in-silico findings.

## Conclusion

The present work offers a distinctive multi-epitope immunization targeting bacteria responsible for intestinal disorders. The efficacy of the suggested vaccine was validated by assessing its robust humoral and cellular immune response using immunoinformatic assessment, protein structure analysis, physicochemical research, and molecular dynamics investigations. These results provide strong evidence that warrants further experimental confirmation.

### Supplementary Information

Supplementary Material 1.

## Data Availability

No datasets were generated or analysed during the current study.
