# Supplementary material for: Putative new combination vaccine candidates identified by reverse vaccinology and genomic approaches to control enteric pathogens
Source: BMC Immunol. 2024 Jul 22;25:46. doi: 10.1186/s12865-024-00626-y (PMC11265179; doi:10.1186/s12865-024-00626-y)
Supplement: Supplementary file 1 — Supplementary Material 1. [file 12865_2024_626_MOESM1_ESM.docx]

**Supplementary Files**

**Table S1.** Genome information for reference (*S. typhimurium* and E. coli) and representative genomes.

| Bacteria | Type |  | Name | RefSeq | INSDC | Size (Mb) | GC% | Protein | rRNA | tRNA | Gene | Pseudogene |
| --- | --- | --- | --- | --- | --- | --- | --- | --- | --- | --- | --- | --- |
| *S. typhimurium* | Chr |  | - | [NC_003197.2](https://www.ncbi.nlm.nih.gov/nuccore/NC_003197.2) | [AE006468.2](https://www.ncbi.nlm.nih.gov/nuccore/AE006468.2) | 4.86 | 52.2 | [4,452](https://www.ncbi.nlm.nih.gov/genome/browse/#!/proteins/152/305903\|Salmonella enterica subsp. enterica serovar Typhimurium str. LT2/chromosome/) | 22 | 85 | 4,608 | 39 |
|  | Plsm |  | pSLT | [NC_003277.2](https://www.ncbi.nlm.nih.gov/nuccore/NC_003277.2) | [AE006471.2](https://www.ncbi.nlm.nih.gov/nuccore/AE006471.2) | 0.09 | 53.1 | [102](https://www.ncbi.nlm.nih.gov/genome/browse/#!/proteins/152/305903\|Salmonella enterica subsp. enterica serovar Typhimurium str. LT2/plasmid pSLT/) | - | - | 109 | - |
| *E. coliO157:H7* | Chr |  | - | [NC_002695.2](https://www.ncbi.nlm.nih.gov/nuccore/NC_002695.2) | [BA000007.3](https://www.ncbi.nlm.nih.gov/nuccore/BA000007.3) | 5.5 | 50.5 | [5,067](https://www.ncbi.nlm.nih.gov/genome/browse/#!/proteins/167/409151\|Escherichia coli O157:H7 str. Sakai/chromosome/) | 22 | 103 | 5,329 | 136 |
|  | Plsm |  | pOSAK1 | [NC_002127.1](https://www.ncbi.nlm.nih.gov/nuccore/NC_002127.1) | [AB011548.2](https://www.ncbi.nlm.nih.gov/nuccore/AB011548.2) | 0 | 43.4 | [3](https://www.ncbi.nlm.nih.gov/genome/browse/#!/proteins/167/409151\|Escherichia coli O157:H7 str. Sakai/plasmid pOSAK1/) | - | - | 3 | - |
|  | Plsm |  | pO157 | [NC_002128.1](https://www.ncbi.nlm.nih.gov/nuccore/NC_002128.1) | [AB011549.2](https://www.ncbi.nlm.nih.gov/nuccore/AB011549.2) | 0.09 | 47.6 | [85](https://www.ncbi.nlm.nih.gov/genome/browse/#!/proteins/167/409151\|Escherichia coli O157:H7 str. Sakai/plasmid pO157/) | - | - | 85 | - |

**Table S2.** Predicted B-cell epitopes from pathogens protein and their corresponding immunogenic properties. The final selected epitopes to design multi-epitope vaccine construct from proteins has been highlighted in green colour.

| Bacteria | ACCESSION NUMBER | Rank | Sequence | Start position | Score |
| --- | --- | --- | --- | --- | --- |
| *S. typhimurium* | AKH09008.1 | 1 | LRQELLLKNS | 80 | 0.84 |
|  |  | 2 | VLLICDATHA | 171 | 0.84 |
|  |  | 3 | PYSDQVVIDK | 130 | 0.83 |
|  |  | 4 | TAVSPFYFIS | 44 | 0.79 |
|  | WP_179714467.1 | 1 | QPILPKAGDT | 45 | 0.73 |
|  |  | 2 | NIQEIPKKEL | 26 | 0.73 |
|  |  | 3 | TVVEVFISED | 9 | 0.65 |
|  |  | 4 | RVQASQTVVE | 3 | 0.65 |
|  | APQ79047.1 | 1 | ANVYSSVQAR | 82 | 0.81 |
|  |  | 2 | GTDATILKNE | 98 | 0.80 |
|  |  | 3 | AAGQTAGDTD | 63 | 0.76 |
|  |  | 4 | KQNACTVKTD | 38 | 0.71 |
|  | QIG55538.1 | 1 | VIPALLAAAT | 7 | 0.72 |
|  |  | 2 | GDSKNADQTY | 46 | 0.72 |
|  |  | 3 | LAAATANAAE | 12 | 0.71 |
|  |  | 4 | NKDGNKLDLY | 24 | 0.68 |
| *E. coliO157:H7* | VWQ00961.1 | 1 | SVGKYSASVK | 143 | 0.83 |
|  |  | 2 | LRREMRQMEG | 365 | 0.80 |
|  |  | 3 | LKLVFQEGVS | 165 | 0.79 |
|  |  | 4 | KDAVLRREMR | 361 | 0.77 |
|  | CAD6011050.1 | 1 | FRLVDSNGSV | 83 | 0.81 |
|  |  | 2 | SNADSYNKKG | 173 | 0.81 |
|  |  | 3 | NMQGLQLTGY | 110 | 0.80 |
|  |  | 4 | VYFVKTGDNN | 198 | 0.78 |
|  | CAD6009997.1 | 1 | PPPEPVVEPE | 68 | 0.80 |
|  |  | 2 | FENTAPARLT | 130 | 0.76 |
|  |  | 3 | PEPEPIPEPP | 78 | 0.75 |
|  |  | 4 | PVESRPASPF | 121 | 0.75 |
|  | ACO48717.1 | 1 | GFINNNGPTH | 3 | 0.85 |
|  |  | 2 | YVGFEMGYDW | 29 | 0.84 |
|  |  | 3 | QVNPYVGFEM | 25 | 0.77 |
|  |  | 4 | ITDDLDIYTR | 68 | 0.76 |
|  | CAD6006050.1 | 1 | WSLWGNASNT | 78 | 0.83 |
|  |  | 2 | KYAAGGRSRF | 97 | 0.79 |
|  |  | 3 | WYGQTTAWSL | 71 | 0.79 |
|  |  | 4 | AQSGNTKSSS | 53 | 0.79 |

**Table S3.** Predicted CTL epitopes from pathogens protein with their corresponding MHC Class I alleles and their immunogenic properties. The final selected epitopes to design multi-epitope vaccine construct from proteins has been highlighted in green color.

---------------------------------------------------------------------------------------------------------------------------

5 HLA-A*01:01 RPSQRHGSKY RSQRHGSKY 0 1 1 0 0 RPSQRHGSKY seq1 0.2172420 0.705 0.222741 1.349 4490.73 <= WB

19 HLA-A*01:01 STMDHARHGF STDHARHGF 0 2 1 0 0 STMDHARHGF seq1 0.1739260 0.841 0.236651 1.196 3863.26 <= WB

78 HLA-A*01:01 RTQDENPVVH RTDENPVVH 0 2 1 0 0 RTQDENPVVH seq1 0.1588880 0.896 0.152085 2.920 9645.55 <= WB

79 HLA-A*01:01 TQDENPVVHF TQDEPVVHF 0 4 1 0 0 TQDENPVVHF seq1 0.0917840 1.359 0.092982 7.582 18283.12 <= WB

79 HLA-A*02:01 TQDENPVVHF TQDEPVVHF 0 4 1 0 0 TQDENPVVHF seq1 0.0473310 2.999 0.091400 24.287 18598.76

54 HLA-A*02:01 NMYKDSHHPA NMYDSHHPA 0 3 1 0 0 NMYKDSHHPA seq1 0.0390620 3.371 0.407407 3.116 608.94

20 HLA-A*02:01 TMDHARHGFL TMDHARHFL 0 7 1 0 0 TMDHARHGFL seq1 0.0340290 3.636 0.355900 4.188 1063.18

28 HLA-A*02:01 FLPRHRDTGI FLPRHDTGI 0 5 1 0 0 FLPRHRDTGI seq1 0.0142800 5.680 0.289836 6.059 2172.90

79 HLA-A*02:01 TQDENPVVHF TQDEPVVHF 0 4 1 0 0 TQDENPVVHF seq1 0.0473310 2.999 0.091400 24.287 18598.76

54 HLA-A*02:01 NMYKDSHHPA NMYDSHHPA 0 3 1 0 0 NMYKDSHHPA seq1 0.0390620 3.371 0.407407 3.116 608.94

20 HLA-A*02:01 TMDHARHGFL TMDHARHFL 0 7 1 0 0 TMDHARHGFL seq1 0.0340290 3.636 0.355900 4.188 1063.18

28 HLA-A*02:01 FLPRHRDTGI FLPRHDTGI 0 5 1 0 0 FLPRHRDTGI seq1 0.0142800 5.680 0.289836 6.059 2172.90

79 HLA-A*02:01 TQDENPVVHF TQDEPVVHF 0 4 1 0 0 TQDENPVVHF seq1 0.0473310 2.999 0.091400 24.287 18598.76

54 HLA-A*02:01 NMYKDSHHPA NMYDSHHPA 0 3 1 0 0 NMYKDSHHPA seq1 0.0390620 3.371 0.407407 3.116 608.94

20 HLA-A*02:01 TMDHARHGFL TMDHARHFL 0 7 1 0 0 TMDHARHGFL seq1 0.0340290 3.636 0.355900 4.188 1063.18

28 HLA-A*02:01 FLPRHRDTGI FLPRHDTGI 0 5 1 0 0 FLPRHRDTGI seq1 0.0142800 5.680 0.289836 6.059 2172.90

77 HLA-A*02:01 VLRDGDTLLV VLDGDTLLV 0 2 1 0 0 VLRDGDTLLV Sequence 0.3200690 0.706 0.524026 1.528 172.42 <= WB

166 HLA-A*02:01 KLVFQEGVSA KLFQEGVSA 0 2 1 0 0 KLVFQEGVSA Sequence 0.2780260 0.829 0.414647 2.982 563.06 <= WB

610 HLA-A*02:01 KVTLDTATYV KVLDTATYV 0 2 1 0 0 KVTLDTATYV Sequence 0.2372700 0.975 0.536904 1.405 149.99 <= WB

238 HLA-A*02:01 FNIDSTQVSL FIDSTQVSL 0 1 1 0 0 FNIDSTQVSL Sequence 0.2175140 1.060 0.417789 2.931 544.24 <= WB

275 HLA-A*03:01 IVATTQNGYK IVATTQNYK 0 7 1 0 0 IVATTQNGYK Sequence 0.3980120 0.639 0.630669 0.218 54.38 <= WB

138 HLA-A*03:01 SIPNTLMAAK SIPNTLMAK 0 7 1 0 0 SIPNTLMAAK Sequence 0.3483830 0.743 0.587737 0.336 86.54 <= WB

172 HLA-A*03:01 ASNADSYNKK ASNDSYNKK 0 3 1 0 0 ASNADSYNKK Sequence 0.3455990 0.749 0.418932 1.219 537.55 <= WB

92 HLA-A*03:01 VFYSRNGQFK VFYSRGQFK 0 5 1 0 0 VFYSRNGQFK Sequence 0.1200380 1.711 0.494742 0.716 236.70 <= WB

174 HLA-A*02:01 ALRIEGQVKV ALIEGQVKV 0 2 1 0 0 ALRIEGQVKV Sequence 0.8751860 0.061 0.610604 0.814 67.57 <= SB

31 HLA-A*02:01 GLLYTSVHQV GLYTSVHQV 0 1 1 0 0 GLLYTSVHQV Sequence 0.6992290 0.181 0.685652 0.440 30.00 <= SB

32 HLA-A*02:01 LLYTSVHQVI LLYTSVHQI 0 8 1 0 0 LLYTSVHQVI Sequence 0.1502850 1.426 0.572660 1.098 101.87 <= WB

28 HLA-A*02:01 VVAGLLYTSV VVAGLLTSV 0 6 1 0 0 VVAGLLYTSV Sequence 0.1354350 1.518 0.596490 0.911 78.72 <= WB

75 HLA-B*27:05 YTRLGGMVWR YRLGGMVWR 0 1 1 0 0 YTRLGGMVWR Sequence 0.3639810 0.694 0.508163 0.628 204.70 <= WB

83 HLA-B*27:05 WRADTKSNVY WRADTKSNY 0 8 1 0 0 WRADTKSNVY Sequence 0.2654270 0.930 0.386816 1.612 760.91 <= WB

136 HLA-B*27:05 TRPDNGMLSL TRDNGMLSL 0 2 1 0 0 TRPDNGMLSL Sequence 0.2039130 1.146 0.249244 4.050 3371.17 <= WB

76 HLA-B*27:05 TRLGGMVWRA TRLGGVWRA 0 5 1 0 0 TRLGGMVWRA Sequence 0.1122350 1.696 0.442899 1.065 414.76 <= WB

145 HLA-B*07:02 GPVHSFRFEF GPHSFRFEF 0 2 1 0 0 GPVHSFRFEF Sequence 0.3762080 0.554 0.532861 0.419 156.70 <= WB

239 HLA-B*07:02 HTDRRTTLSL HTRRTTLSL 0 2 1 0 0 HTDRRTTLSL Sequence 0.1302710 1.211 0.478246 0.596 282.95 <= WB

236 HLA-B*07:02 APEHTDRRTT APHTDRRTT 0 2 1 0 0 APEHTDRRTT Sequence 0.1124810 1.351 0.332424 1.518 1370.63 <= WB

40 HLA-B*07:02 KKPFEGNLNA KPFEGNLNA 0 1 1 0 0 KKPFEGNLNA Sequence 0.0544120 2.125 0.228933 3.085 4199.72

**Table S4.** Predicted HTL epitopes from *pathogens* protein with their corresponding MHC Class II alleles and their immunogenic properties. The final selected epitopes to design multi-epitope vaccine construct from proteins has been highlighted in green color.

| Bacteria | ACCESSION NUMBER | Rank | Allele | Sequence | Start position | percentile_rank |
| --- | --- | --- | --- | --- | --- | --- |
| *S. typhimurium* | AKH09008.1 | 1 | HLA-DRB1*01:01 | SLQIRLILAVLVALG | 10 | 0.01 |
|  |  | 2 | HLA-DRB3*02:02 | PFYFISNGPRELLDS | 48 | 0.11 |
|  |  | 3 | HLA-DRB1*03:01 | VSSVKLDTQRAYTVI | 28 | 1.30 |
|  |  | 4 | HLA-DRB1*13:02 | PSLQIRLILAVLVAL | 9 | 5.20 |
|  | WP_179714467.1 | 1 | HLA-DRB1*01:01 | SLQIRLILAVLVALG | 10 | 0.01 |
|  |  | 2 | HLA-DRB3*02:02 | FYFISNGPRELL | 49 | 0.03 |
|  |  | 3 | HLA-DRB1*09:01 | PIFSRGPSLQIRLIL | 3 | 0.24 |
|  |  | 4 | HLA-DRB1*03:01 | LVALGVIIADSRLGT | 20 | 0.53 |
|  | APQ79047.1 | 1 | HLA-DPA1*02:01/DPB1*14:01 | ARYIATAVPVKS | 14 | 0.04 |
|  |  | 2 | \| HLA-DQA1*03:01/DQB1*03:02 \|  \| \| --- \| --- \| | TDSVEVTLQEEF | 46 | 0.07 |
|  |  | 3 | HLA-DRB1*01:01 | QEEFASLFTAAG | 54 | 1.40 |
|  |  | 4 | HLA-DQA1*03:01/DQB1*03:02 | DDAENIGVQILD | 38 | 1.60 |
|  | QIG55538.1 | 1 | HLA-DPA1*02:01/DPB1*14:01 | KILAAVIPALLA | 2 | 0.03 |
|  |  | 2 | HLA-DQA1*05:01/DQB1*03:01 | ALLAAATANAAE | 10 | 0.18 |
|  |  | 3 | HLA-DRB1*07:01 | LDLYGKAVGRHV | 30 | 2.20 |
|  |  | 4 | HLA-DRB5*01:01 | LDLYGKAVGRHV | 30 | 6.20 |
| *E. coliO157:H7* | VWQ00961.1 | 1 | HLA-DRB5*01:01 | LVFSYAQPFKKY | 13 | 0.01 |
|  |  | 2 | HLA-DRB3*01:01 | RSYYLDRGYARF | 17 | 0.19 |
|  |  | 3 | HLA-DRB1*04:05 | AMWRYLYSMGEH | 54 | 0.35 |
|  |  | 4 | HLA-DRB3*01:01 | YARFNIDSTQVS | 25 | 0.48 |
|  | CAD6011050.1 | 1 | HLA-DRB1*04:05 | NGFFRLVDSNGS | 10 | 0.11 |
|  |  | 2 | HLA-DRB3*01:01 | SNADSYNKKG | 28 | 0.18 |
|  |  | 3 | HLA-DQA1*05:01/DQB1*03:01 | NMQGLQLTGY | 33 | 0.20 |
|  |  | 4 | HLA-DRB4*01:01 | KELVNMIVAQRN | 19 | 0.73 |
|  | CAD6009997.1 | 1 | HLA-DRB3*02:02 | ILFKINGTTEIQ | 23 | 0.05 |
|  |  | 2 | HLA-DRB3*01:01 | MTSMTLDLPRRF | 3 | 0.13 |
|  |  | 3 | HLA-DRB1*09:01 | SPFENTAPARLT | 58 | 0.43 |
|  |  | 4 | HLA-DRB5*01:01 | EAPVVIEKPKPK | 19 | 0.60 |
|  | ACO48717.1 | 1 | HLA-DQA1*05:01/DQB1*03:01 | SPVFAGGVEYAI | 31 | 0.54 |
|  |  | 2 | HLA-DRB3*02:02 | TGFINNNGPTHE | 2 | 0.94 |
|  |  | 3 | HLA-DQA1*03:01/DQB1*03:02 | GGVEYAITPEIA | 36 | 1.9 |
|  |  | 4 | HLA-DRB3*01:01 | MVWRADTKSNVY | 11 | 2.30 |
|  | CAD6006050.1 | 1 | HLA-DRB1*01:01 | MKLLKTVPAIVM | 1 | 0.02 |
|  |  | 2 | HLA-DRB1*09:01 | GGMFASLNAAAD | 15 | 1.20 |
|  |  | 3 | HLA-DRB3*01:01 | QASWLTDRYNGY | 47 | 1.60 |
|  |  | 4 | HLA-DRB3*02:02 | GLKVAYNVTWNS | 9 | 1.70 |

**Table S5.** MolProbity Results of Ramachandran plot.

| MolProbity Score | 0.90 |  |
| --- | --- | --- |
| Clash Score | 1.54 |  |
| Ramachandran Favoured | 100.00% |  |
| Ramachandran Outliers | 0.00% |  |
| Rotamer Outliers | 0.00% |  |
| C-Beta Deviations | 1 | D80 ILE |
| Bad Bonds | 0 / 315 |  |
| Bad Angles | 2 / 424 | (D80 ILE-D81 PRO), D104 HIS |

**Table S6.** Alignment Cluster 1 for chosen vaccine model.

NUMMDL 1

REMARK Alignment Cluster 1

REMARK Partial threaded model 8jncA_201

MODEL 1

ATOM 1 N SER A 8 -15.341 11.713 -8.249 1.00 0.00 N

ATOM 2 CA SER A 8 -16.194 12.132 -9.360 1.00 0.00 C

ATOM 3 C SER A 8 -15.521 11.809 -10.696 1.00 0.00 C

ATOM 4 O SER A 8 -14.320 12.028 -10.857 1.00 0.00 O

ATOM 12 N PRO A 9 -16.277 11.237 -11.652 1.00 0.00 N

ATOM 13 CA PRO A 9 -15.621 10.790 -12.887 1.00 0.00 C

ATOM 14 C PRO A 9 -15.125 11.943 -13.731 1.00 0.00 C

ATOM 15 O PRO A 9 -14.352 11.720 -14.676 1.00 0.00 O

ATOM 26 N SER A 10 -9.947 13.042 -13.665 1.00 0.00 N

ATOM 27 CA SER A 10 -9.102 11.965 -14.124 1.00 0.00 C

ATOM 28 C SER A 10 -7.762 12.033 -13.418 1.00 0.00 C

ATOM 29 O SER A 10 -7.239 13.126 -13.179 1.00 0.00 O

ATOM 37 N VAL A 11 -7.179 10.865 -13.149 1.00 0.00 N

ATOM 38 CA VAL A 11 -5.824 10.793 -12.599 1.00 0.00 C

ATOM 39 C VAL A 11 -4.773 10.815 -13.692 1.00 0.00 C

ATOM 40 O VAL A 11 -3.580 10.788 -13.381 1.00 0.00 O

ATOM 53 N ARG A 12 -5.206 10.890 -14.957 1.00 0.00 N

ATOM 54 CA ARG A 12 -4.346 10.838 -16.143 1.00 0.00 C

ATOM 55 C ARG A 12 -4.222 12.222 -16.781 1.00 0.00 C

ATOM 56 O ARG A 12 -5.225 12.865 -17.088 1.00 0.00 O

ATOM 77 N HIS A 13 -2.989 12.657 -17.044 1.00 0.00 N

ATOM 78 CA HIS A 13 -2.764 13.939 -17.728 1.00 0.00 C

ATOM 79 C HIS A 13 -2.826 13.675 -19.235 1.00 0.00 C

ATOM 80 O HIS A 13 -1.809 13.560 -19.903 1.00 0.00 O

ATOM 94 N GLU A 14 -4.033 13.554 -19.774 1.00 0.00 N

ATOM 95 CA GLU A 14 -4.256 13.243 -21.192 1.00 0.00 C

ATOM 96 C GLU A 14 -4.283 14.489 -22.092 1.00 0.00 C

ATOM 97 O GLU A 14 -4.552 14.391 -23.309 1.00 0.00 O

ATOM 109 N ALA A 15 -4.009 15.660 -21.515 1.00 0.00 N

ATOM 110 CA ALA A 15 -4.035 16.920 -22.240 1.00 0.00 C

ATOM 111 C ALA A 15 -2.681 17.603 -22.252 1.00 0.00 C

ATOM 112 O ALA A 15 -2.612 18.822 -22.383 1.00 0.00 O

ATOM 119 N ALA A 16 -1.605 16.854 -22.118 1.00 0.00 N

ATOM 120 CA ALA A 16 -0.288 17.419 -22.184 1.00 0.00 C

ATOM 121 C ALA A 16 0.214 18.035 -20.909 1.00 0.00 C

ATOM 122 O ALA A 16 1.341 18.543 -20.912 1.00 0.00 O

ATOM 129 N ALA A 17 -0.554 17.965 -19.812 1.00 0.00 N

ATOM 130 CA ALA A 17 -0.139 18.582 -18.555 1.00 0.00 C

ATOM 131 C ALA A 17 1.196 18.041 -18.035 1.00 0.00 C

ATOM 132 O ALA A 17 1.545 16.875 -18.199 1.00 0.00 O

ATOM 139 N LYS A 18 1.910 18.904 -17.340 1.00 0.00 N

ATOM 140 CA LYS A 18 3.120 18.542 -16.621 1.00 0.00 C

ATOM 141 C LYS A 18 2.696 18.306 -15.175 1.00 0.00 C

ATOM 142 O LYS A 18 2.317 19.240 -14.474 1.00 0.00 O

ATOM 161 N PRO A 19 2.755 17.053 -14.733 1.00 0.00 N

ATOM 162 CA PRO A 19 2.156 16.628 -13.471 1.00 0.00 C

ATOM 163 C PRO A 19 2.596 17.469 -12.276 1.00 0.00 C

ATOM 164 O PRO A 19 3.796 17.480 -11.941 1.00 0.00 O

ATOM 175 N PRO A 20 1.673 18.165 -11.609 1.00 0.00 N

ATOM 176 CA PRO A 20 2.044 18.890 -10.390 1.00 0.00 C

ATOM 177 C PRO A 20 2.580 17.901 -9.364 1.00 0.00 C

ATOM 178 O PRO A 20 1.951 16.882 -9.084 1.00 0.00 O

ATOM 189 N PRO A 21 3.772 18.181 -8.823 1.00 0.00 N

ATOM 190 CA PRO A 21 4.428 17.175 -7.998 1.00 0.00 C

ATOM 191 C PRO A 21 5.516 17.785 -7.129 1.00 0.00 C

ATOM 192 O PRO A 21 5.892 18.939 -7.293 1.00 0.00 O

ATOM 203 N GLU A 22 6.000 16.976 -6.184 1.00 0.00 N

ATOM 204 CA GLU A 22 7.093 17.337 -5.292 1.00 0.00 C

ATOM 205 C GLU A 22 8.373 16.571 -5.619 1.00 0.00 C

ATOM 206 O GLU A 22 9.444 16.899 -5.079 1.00 0.00 O

ATOM 218 N PRO A 23 8.289 15.575 -6.501 1.00 0.00 N

ATOM 219 CA PRO A 23 9.418 14.745 -6.906 1.00 0.00 C

ATOM 220 C PRO A 23 9.857 15.129 -8.307 1.00 0.00 C

ATOM 221 O PRO A 23 9.029 15.286 -9.208 1.00 0.00 O

ATOM 232 N VAL A 24 11.171 15.271 -8.491 1.00 0.00 N

ATOM 233 CA VAL A 24 11.685 15.751 -9.769 1.00 0.00 C

ATOM 234 C VAL A 24 11.207 14.873 -10.918 1.00 0.00 C

ATOM 235 O VAL A 24 10.789 15.382 -11.959 1.00 0.00 O

ATOM 248 N VAL A 25 11.222 13.564 -10.736 1.00 0.00 N

ATOM 249 CA VAL A 25 10.719 12.660 -11.751 1.00 0.00 C

ATOM 250 C VAL A 25 9.227 12.383 -11.649 1.00 0.00 C

ATOM 251 O VAL A 25 8.770 11.871 -10.624 1.00 0.00 O

ATOM 264 N GLU A 26 8.482 12.678 -12.720 1.00 0.00 N

ATOM 265 CA GLU A 26 7.040 12.465 -12.780 1.00 0.00 C

ATOM 266 C GLU A 26 6.683 11.019 -12.459 1.00 0.00 C

ATOM 267 O GLU A 26 7.423 10.079 -12.745 1.00 0.00 O

ATOM 279 N PRO A 27 5.529 10.851 -11.840 1.00 0.00 N

ATOM 280 CA PRO A 27 5.134 9.562 -11.291 1.00 0.00 C

ATOM 281 C PRO A 27 4.353 8.882 -12.385 1.00 0.00 C

ATOM 282 O PRO A 27 3.186 9.194 -12.601 1.00 0.00 O

ATOM 293 N GLU A 28 4.995 7.964 -13.096 1.00 0.00 N

ATOM 294 CA GLU A 28 4.375 7.358 -14.267 1.00 0.00 C

ATOM 295 C GLU A 28 4.696 5.875 -14.349 1.00 0.00 C

ATOM 296 O GLU A 28 5.791 5.435 -13.999 1.00 0.00 O

ATOM 308 N LYS A 29 3.724 5.097 -14.796 1.00 0.00 N

ATOM 309 CA LYS A 29 3.934 3.674 -14.895 1.00 0.00 C

ATOM 310 C LYS A 29 2.671 3.026 -15.383 1.00 0.00 C

ATOM 311 O LYS A 29 1.793 3.710 -15.903 1.00 0.00 O

ATOM 330 N LYS A 30 2.574 1.711 -15.174 1.00 0.00 N

ATOM 331 CA LYS A 30 1.518 0.872 -15.725 1.00 0.00 C

ATOM 332 C LYS A 30 0.598 0.268 -14.670 1.00 0.00 C

ATOM 333 O LYS A 30 -0.198 -0.634 -14.999 1.00 0.00 O

ATOM 352 N GLY A 31 0.691 0.745 -13.432 1.00 0.00 N

ATOM 353 CA GLY A 31 -0.078 0.233 -12.314 1.00 0.00 C

ATOM 354 C GLY A 31 -1.319 1.053 -11.991 1.00 0.00 C

ATOM 355 O GLY A 31 -1.805 1.846 -12.804 1.00 0.00 O

ATOM 359 N PHE A 32 -1.804 0.906 -10.746 1.00 0.00 N

ATOM 360 CA PHE A 32 -3.176 1.300 -10.430 1.00 0.00 C

ATOM 361 C PHE A 32 -3.365 2.808 -10.434 1.00 0.00 C

ATOM 362 O PHE A 32 -4.452 3.290 -10.772 1.00 0.00 O

ATOM 379 N ILE A 33 -2.324 3.552 -10.068 1.00 0.00 N

ATOM 380 CA ILE A 33 -2.322 5.015 -10.026 1.00 0.00 C

ATOM 381 C ILE A 33 -2.188 5.647 -11.416 1.00 0.00 C

ATOM 382 O ILE A 33 -2.314 6.875 -11.567 1.00 0.00 O

ATOM 398 N ASN A 34 -1.957 4.859 -12.447 1.00 0.00 N

ATOM 399 CA ASN A 34 -1.837 5.390 -13.806 1.00 0.00 C

ATOM 400 C ASN A 34 -3.134 5.279 -14.615 1.00 0.00 C

ATOM 401 O ASN A 34 -3.163 5.663 -15.791 1.00 0.00 O

ATOM 412 N ASN A 35 -4.205 4.774 -14.011 1.00 0.00 N

ATOM 413 CA ASN A 35 -5.423 4.437 -14.736 1.00 0.00 C

ATOM 414 C ASN A 35 -6.671 5.015 -14.094 1.00 0.00 C

ATOM 415 O ASN A 35 -6.779 5.100 -12.862 1.00 0.00 O

ATOM 426 N ASN A 36 -7.647 5.382 -14.979 1.00 0.00 N

ATOM 427 CA ASN A 36 -9.025 5.673 -14.640 1.00 0.00 C

ATOM 428 C ASN A 36 -9.923 4.443 -14.883 1.00 0.00 C

ATOM 429 O ASN A 36 -9.589 3.564 -15.675 1.00 0.00 O

ATOM 440 N GLY A 37 -11.087 4.367 -14.236 1.00 0.00 N

ATOM 441 CA GLY A 37 -12.076 3.357 -14.632 1.00 0.00 C

ATOM 442 C GLY A 37 -12.491 3.558 -16.056 1.00 0.00 C

ATOM 443 O GLY A 37 -12.432 4.685 -16.598 1.00 0.00 O

ATOM 447 N PRO A 38 -12.887 2.497 -16.761 1.00 0.00 N

ATOM 448 CA PRO A 38 -12.970 1.093 -16.317 1.00 0.00 C

ATOM 449 C PRO A 38 -11.664 0.336 -16.347 1.00 0.00 C

ATOM 450 O PRO A 38 -11.555 -0.739 -15.771 1.00 0.00 O

ATOM 461 N THR A 39 -10.690 0.868 -17.082 1.00 0.00 N

ATOM 462 CA THR A 39 -9.421 0.170 -17.175 1.00 0.00 C

ATOM 463 C THR A 39 -8.842 -0.038 -15.784 1.00 0.00 C

ATOM 464 O THR A 39 -8.392 -1.117 -15.458 1.00 0.00 O

ATOM 475 N HIS A 40 -8.858 0.979 -14.918 1.00 0.00 N

ATOM 476 CA HIS A 40 -8.352 0.759 -13.586 1.00 0.00 C

ATOM 477 C HIS A 40 -9.131 -0.363 -12.919 1.00 0.00 C

ATOM 478 O HIS A 40 -8.603 -1.248 -12.240 1.00 0.00 O

ATOM 492 N LYS A 41 -10.429 -0.326 -13.046 1.00 0.00 N

ATOM 493 CA LYS A 41 -11.222 -1.292 -12.324 1.00 0.00 C

ATOM 494 C LYS A 41 -10.907 -2.699 -12.782 1.00 0.00 C

ATOM 495 O LYS A 41 -11.014 -3.644 -12.009 1.00 0.00 O

ATOM 514 N LYS A 42 -10.538 -2.898 -14.043 1.00 0.00 N

ATOM 515 CA LYS A 42 -10.301 -4.253 -14.524 1.00 0.00 C

ATOM 516 C LYS A 42 -8.963 -4.770 -14.021 1.00 0.00 C

ATOM 517 O LYS A 42 -8.861 -5.906 -13.532 1.00 0.00 O

ATOM 536 N LEU A 43 -7.977 -3.887 -13.925 1.00 0.00 N

ATOM 537 CA LEU A 43 -6.672 -4.249 -13.374 1.00 0.00 C

ATOM 538 C LEU A 43 -6.771 -4.499 -11.884 1.00 0.00 C

ATOM 539 O LEU A 43 -6.216 -5.481 -11.384 1.00 0.00 O

ATOM 555 N ARG A 44 -7.467 -3.621 -11.155 1.00 0.00 N

ATOM 556 CA ARG A 44 -7.574 -3.807 -9.722 1.00 0.00 C

ATOM 557 C ARG A 44 -8.297 -5.106 -9.401 1.00 0.00 C

ATOM 558 O ARG A 44 -7.984 -5.780 -8.417 1.00 0.00 O

ATOM 579 N GLN A 45 -9.282 -5.464 -10.210 1.00 0.00 N

ATOM 580 CA GLN A 45 -10.044 -6.673 -9.911 1.00 0.00 C

ATOM 581 C GLN A 45 -9.139 -7.909 -9.806 1.00 0.00 C

ATOM 582 O GLN A 45 -9.521 -8.897 -9.167 1.00 0.00 O

ATOM 596 N GLU A 46 -7.945 -7.887 -10.402 1.00 0.00 N

ATOM 597 CA GLU A 46 -7.056 -9.040 -10.274 1.00 0.00 C

ATOM 598 C GLU A 46 -6.611 -9.240 -8.826 1.00 0.00 C

ATOM 599 O GLU A 46 -6.366 -10.378 -8.382 1.00 0.00 O

ATOM 611 N LEU A 47 -6.474 -8.144 -8.075 1.00 0.00 N

ATOM 612 CA LEU A 47 -6.099 -8.198 -6.671 1.00 0.00 C

ATOM 613 C LEU A 47 -7.273 -8.190 -5.709 1.00 0.00 C

ATOM 614 O LEU A 47 -7.089 -8.516 -4.526 1.00 0.00 O

ATOM 630 N LEU A 48 -8.443 -7.741 -6.169 1.00 0.00 N

ATOM 631 CA LEU A 48 -9.540 -7.356 -5.305 1.00 0.00 C

ATOM 632 C LEU A 48 -9.824 -8.309 -4.171 1.00 0.00 C

ATOM 633 O LEU A 48 -9.803 -7.920 -2.982 1.00 0.00 O

ATOM 649 N LEU A 49 -10.056 -9.577 -4.561 1.00 0.00 N

ATOM 650 CA LEU A 49 -10.500 -10.602 -3.626 1.00 0.00 C

ATOM 651 C LEU A 49 -9.538 -10.770 -2.462 1.00 0.00 C

ATOM 652 O LEU A 49 -9.972 -11.114 -1.360 1.00 0.00 O

ATOM 668 N LYS A 50 -8.253 -10.491 -2.651 1.00 0.00 N

ATOM 669 CA LYS A 50 -7.288 -10.767 -1.588 1.00 0.00 C

ATOM 670 C LYS A 50 -7.242 -9.696 -0.501 1.00 0.00 C

ATOM 671 O LYS A 50 -6.568 -9.917 0.530 1.00 0.00 O

ATOM 690 N ASN A 51 -7.932 -8.559 -0.708 1.00 0.00 N

ATOM 691 CA ASN A 51 -7.930 -7.431 0.230 1.00 0.00 C

ATOM 692 C ASN A 51 -9.315 -7.101 0.800 1.00 0.00 C

ATOM 693 O ASN A 51 -9.436 -6.178 1.623 1.00 0.00 O

ATOM 704 N SER A 52 -10.332 -7.895 0.499 1.00 0.00 N

ATOM 705 CA SER A 52 -11.630 -7.720 1.157 1.00 0.00 C

ATOM 706 C SER A 52 -11.536 -7.980 2.658 1.00 0.00 C

ATOM 707 O SER A 52 -10.675 -8.738 3.104 1.00 0.00 O

ATOM 715 N PRO A 59 -12.434 -7.359 3.466 1.00 0.00 N

ATOM 716 CA PRO A 59 -12.410 -7.652 4.919 1.00 0.00 C

ATOM 717 C PRO A 59 -12.498 -9.161 5.226 1.00 0.00 C

ATOM 718 O PRO A 59 -11.848 -9.633 6.169 1.00 0.00 O

ATOM 729 N LYS A 60 -13.240 -9.938 4.435 1.00 0.00 N

ATOM 730 CA LYS A 60 -13.406 -11.364 4.722 1.00 0.00 C

ATOM 731 C LYS A 60 -12.087 -12.136 4.607 1.00 0.00 C

ATOM 732 O LYS A 60 -11.866 -13.083 5.369 1.00 0.00 O

ATOM 751 N ALA A 61 -11.198 -11.739 3.684 1.00 0.00 N

ATOM 752 CA ALA A 61 -9.919 -12.417 3.521 1.00 0.00 C

ATOM 753 C ALA A 61 -8.876 -11.849 4.478 1.00 0.00 C

ATOM 754 O ALA A 61 -8.192 -12.596 5.178 1.00 0.00 O

ATOM 761 N VAL A 69 -8.794 -10.530 4.571 1.00 0.00 N

ATOM 762 CA VAL A 69 -7.852 -9.892 5.485 1.00 0.00 C

ATOM 763 C VAL A 69 -8.126 -10.206 6.965 1.00 0.00 C

ATOM 764 O VAL A 69 -7.191 -10.216 7.784 1.00 0.00 O

ATOM 777 N TYR A 70 -9.396 -10.396 7.361 1.00 0.00 N

ATOM 778 CA TYR A 70 -9.669 -10.702 8.762 1.00 0.00 C

ATOM 779 C TYR A 70 -8.976 -12.016 9.137 1.00 0.00 C

ATOM 780 O TYR A 70 -8.611 -12.215 10.297 1.00 0.00 O

ATOM 798 N SER A 71 -8.719 -12.883 8.147 1.00 0.00 N

ATOM 799 CA SER A 71 -8.063 -14.168 8.397 1.00 0.00 C

ATOM 800 C SER A 71 -6.608 -13.999 8.817 1.00 0.00 C

ATOM 801 O SER A 71 -6.030 -14.915 9.406 1.00 0.00 O

ATOM 809 N SER A 72 -5.995 -12.859 8.485 1.00 0.00 N

ATOM 810 CA SER A 72 -4.596 -12.568 8.792 1.00 0.00 C

ATOM 811 C SER A 72 -4.360 -11.979 10.190 1.00 0.00 C

ATOM 812 O SER A 72 -3.203 -11.795 10.586 1.00 0.00 O

ATOM 820 N VAL A 73 -5.419 -11.669 10.938 1.00 0.00 N

ATOM 821 CA VAL A 73 -5.261 -11.112 12.275 1.00 0.00 C

ATOM 822 C VAL A 73 -4.306 -11.949 13.135 1.00 0.00 C

ATOM 823 O VAL A 73 -3.429 -11.410 13.812 1.00 0.00 O

ATOM 836 N GLN A 74 -4.477 -13.270 13.155 1.00 0.00 N

ATOM 837 CA GLN A 74 -3.637 -14.089 14.042 1.00 0.00 C

ATOM 838 C GLN A 74 -2.168 -14.017 13.641 1.00 0.00 C

ATOM 839 O GLN A 74 -1.294 -13.860 14.495 1.00 0.00 O

ATOM 853 N ALA A 75 -1.877 -14.066 12.334 1.00 0.00 N

ATOM 854 CA ALA A 75 -0.494 -13.941 11.863 1.00 0.00 C

ATOM 855 C ALA A 75 0.089 -12.562 12.169 1.00 0.00 C

ATOM 856 O ALA A 75 1.229 -12.451 12.639 1.00 0.00 O

ATOM 863 N ARG A 76 -0.706 -11.502 11.994 1.00 0.00 N

ATOM 864 CA ARG A 76 -0.209 -10.142 12.251 1.00 0.00 C

ATOM 865 C ARG A 76 0.214 -10.004 13.709 1.00 0.00 C

ATOM 866 O ARG A 76 1.270 -9.416 14.025 1.00 0.00 O

ATOM 887 N LYS A 77 -0.585 -10.577 14.612 1.00 0.00 N

ATOM 888 CA LYS A 77 -0.247 -10.583 16.034 1.00 0.00 C

ATOM 889 C LYS A 77 0.975 -11.450 16.326 1.00 0.00 C

ATOM 890 O LYS A 77 1.807 -11.068 17.144 1.00 0.00 O

ATOM 909 N LYS A 78 1.107 -12.612 15.678 1.00 0.00 N

ATOM 910 CA LYS A 78 2.303 -13.434 15.870 1.00 0.00 C

ATOM 911 C LYS A 78 3.568 -12.612 15.599 1.00 0.00 C

ATOM 912 O LYS A 78 4.467 -12.521 16.449 1.00 0.00 O

ATOM 931 N VAL A 79 3.612 -11.963 14.430 1.00 0.00 N

ATOM 932 CA VAL A 79 4.765 -11.157 14.025 1.00 0.00 C

ATOM 933 C VAL A 79 4.971 -9.970 14.944 1.00 0.00 C

ATOM 934 O VAL A 79 6.104 -9.633 15.305 1.00 0.00 O

ATOM 947 N ILE A 80 3.880 -9.293 15.300 1.00 0.00 N

ATOM 948 CA ILE A 80 3.954 -8.210 16.272 1.00 0.00 C

ATOM 949 C ILE A 80 4.559 -8.710 17.590 1.00 0.00 C

ATOM 950 O ILE A 80 5.429 -8.045 18.168 1.00 0.00 O

ATOM 966 N PRO A 81 4.141 -9.900 18.057 1.00 0.00 N

ATOM 967 CA PRO A 81 4.687 -10.456 19.298 1.00 0.00 C

ATOM 968 C PRO A 81 6.165 -10.778 19.178 1.00 0.00 C

ATOM 969 O PRO A 81 6.941 -10.509 20.102 1.00 0.00 O

ATOM 980 N ALA A 82 6.561 -11.376 18.060 1.00 0.00 N

ATOM 981 CA ALA A 82 7.971 -11.639 17.831 1.00 0.00 C

ATOM 982 C ALA A 82 8.769 -10.352 17.821 1.00 0.00 C

ATOM 983 O ALA A 82 9.858 -10.300 18.385 1.00 0.00 O

ATOM 990 N LEU A 83 8.220 -9.283 17.222 1.00 0.00 N

ATOM 991 CA LEU A 83 8.926 -8.004 17.194 1.00 0.00 C

ATOM 992 C LEU A 83 9.120 -7.468 18.604 1.00 0.00 C

ATOM 993 O LEU A 83 10.195 -6.978 18.957 1.00 0.00 O

ATOM 1009 N LEU A 84 8.084 -7.576 19.432 1.00 0.00 N

ATOM 1010 CA LEU A 84 8.139 -7.002 20.768 1.00 0.00 C

ATOM 1011 C LEU A 84 9.001 -7.844 21.709 1.00 0.00 C

ATOM 1012 O LEU A 84 9.592 -7.299 22.644 1.00 0.00 O

ATOM 1028 N ALA A 85 9.072 -9.161 21.493 1.00 0.00 N

ATOM 1029 CA ALA A 85 10.016 -9.979 22.258 1.00 0.00 C

ATOM 1030 C ALA A 85 11.440 -9.490 22.024 1.00 0.00 C

ATOM 1031 O ALA A 85 12.203 -9.278 22.967 1.00 0.00 O

ATOM 1038 N ALA A 86 11.806 -9.285 20.755 1.00 0.00 N

ATOM 1039 CA ALA A 86 13.142 -8.781 20.437 1.00 0.00 C

ATOM 1040 C ALA A 86 13.347 -7.370 20.980 1.00 0.00 C

ATOM 1041 O ALA A 86 14.418 -7.056 21.523 1.00 0.00 O

ATOM 1048 N ALA A 87 12.347 -6.499 20.837 1.00 0.00 N

ATOM 1049 CA ALA A 87 12.460 -5.167 21.436 1.00 0.00 C

ATOM 1050 C ALA A 87 12.754 -5.283 22.914 1.00 0.00 C

ATOM 1051 O ALA A 87 13.687 -4.668 23.429 1.00 0.00 O

ATOM 1058 N THR A 88 11.952 -6.077 23.614 1.00 0.00 N

ATOM 1059 CA THR A 88 12.083 -6.167 25.064 1.00 0.00 C

ATOM 1060 C THR A 88 13.418 -6.784 25.449 1.00 0.00 C

ATOM 1061 O THR A 88 14.048 -6.346 26.416 1.00 0.00 O

ATOM 1072 N LYS A 89 13.883 -7.777 24.671 1.00 0.00 N

ATOM 1073 CA LYS A 89 15.190 -8.389 24.899 1.00 0.00 C

ATOM 1074 C LYS A 89 16.319 -7.382 24.699 1.00 0.00 C

ATOM 1075 O LYS A 89 17.382 -7.501 25.322 1.00 0.00 O

ATOM 1094 N LYS A 90 15.140 -1.471 27.645 1.00 0.00 N

ATOM 1095 CA LYS A 90 14.630 -0.117 27.712 1.00 0.00 C

ATOM 1096 C LYS A 90 15.739 0.895 27.997 1.00 0.00 C

ATOM 1097 O LYS A 90 16.554 0.698 28.902 1.00 0.00 O

ATOM 1116 N LYS A 91 15.758 2.015 27.254 1.00 0.00 N

ATOM 1117 CA LYS A 91 14.825 2.417 26.191 1.00 0.00 C

ATOM 1118 C LYS A 91 15.230 1.819 24.860 1.00 0.00 C

ATOM 1119 O LYS A 91 16.414 1.688 24.644 1.00 0.00 O

ATOM 1138 N LEU A 92 14.268 1.435 24.009 1.00 0.00 N

ATOM 1139 CA LEU A 92 14.555 0.846 22.711 1.00 0.00 C

ATOM 1140 C LEU A 92 13.793 1.545 21.584 1.00 0.00 C

ATOM 1141 O LEU A 92 12.786 2.217 21.812 1.00 0.00 O

ATOM 1157 N VAL A 93 14.288 1.374 20.352 1.00 0.00 N

ATOM 1158 CA VAL A 93 13.738 2.062 19.178 1.00 0.00 C

ATOM 1159 C VAL A 93 12.545 1.281 18.643 1.00 0.00 C

ATOM 1160 O VAL A 93 12.701 0.203 18.068 1.00 0.00 O

ATOM 1173 N PHE A 94 11.341 1.808 18.857 1.00 0.00 N

ATOM 1174 CA PHE A 94 10.168 1.114 18.347 1.00 0.00 C

ATOM 1175 C PHE A 94 10.138 1.097 16.815 1.00 0.00 C

ATOM 1176 O PHE A 94 9.536 0.202 16.222 1.00 0.00 O

ATOM 1193 N GLN A 95 10.813 2.033 16.160 1.00 0.00 N

ATOM 1194 CA GLN A 95 10.815 2.048 14.701 1.00 0.00 C

ATOM 1195 C GLN A 95 11.580 0.858 14.168 1.00 0.00 C

ATOM 1196 O GLN A 95 11.077 0.120 13.312 1.00 0.00 O

ATOM 1210 N GLU A 96 12.816 0.660 14.646 1.00 0.00 N

ATOM 1211 CA GLU A 96 13.609 -0.458 14.138 1.00 0.00 C

ATOM 1212 C GLU A 96 13.065 -1.806 14.603 1.00 0.00 C

ATOM 1213 O GLU A 96 13.136 -2.788 13.859 1.00 0.00 O

ATOM 1225 N GLY A 97 12.521 -1.892 15.812 1.00 0.00 N

ATOM 1226 CA GLY A 97 12.107 -3.204 16.328 1.00 0.00 C

ATOM 1227 C GLY A 97 10.695 -3.623 15.938 1.00 0.00 C

ATOM 1228 O GLY A 97 10.381 -4.821 15.981 1.00 0.00 O

ATOM 1232 N VAL A 98 9.824 -2.680 15.582 1.00 0.00 N

ATOM 1233 CA VAL A 98 8.451 -3.071 15.255 1.00 0.00 C

ATOM 1234 C VAL A 98 7.906 -2.390 13.994 1.00 0.00 C

ATOM 1235 O VAL A 98 7.392 -3.067 13.096 1.00 0.00 O

ATOM 1248 N SER A 99 7.989 -1.061 13.929 1.00 0.00 N

ATOM 1249 CA SER A 99 7.267 -0.339 12.886 1.00 0.00 C

ATOM 1250 C SER A 99 7.760 -0.767 11.518 1.00 0.00 C

ATOM 1251 O SER A 99 6.957 -0.964 10.587 1.00 0.00 O

TER

ENDMDL
